# Supplementary material for: The pseudoknot region and poly-(C) tract comprise an essential RNA packaging signal for assembly of foot-and-mouth disease virus
Source: PLoS Pathog. 2024 Dec 23;20(12):e1012283. doi: 10.1371/journal.ppat.1012283 (PMC11734982; doi:10.1371/journal.ppat.1012283)
Supplement: S1 Fig — (A) Replicon GFP expression and trans-encapsidation efficiency in the presence of an RNA replication inhibitor. The ΔP1 and ΔLbdcap GFP replicons were transfected into cells with (green and orange lines) and without (purple and black lines) the presence of GuHCl, a replication inhibitor. The green MFI was read over time using the Incucyte S3 Live cell imager to measure GFP expression levels from both replicating RNA and from input-only translation. The data shown represent the mean from triplicate wells, and the error bars represent the SEM from 12 images. (B) Replicon GFP expression and trans-encapsidation efficiency in the presence of an RNA replication inhibitor. The readout of the second round of the trans-encapsidation assay, measured by counting the number of GFP foci, when the first round was carried out in the presence or absence of GuHCl. The data shown represent the mean from triplicate wells, and the error bars represent the SEM from 12 images. (C, D) Mean and Standard Error data for S1 Fig A and B. (E) Representative images analysed using the Incucyte software to obtain the: MFI data at the point of harvest for: (i-vi) S1 Fig A ΔP1 and ΔLbdcap GFP replicons, in the presence and absence of GuHCl, and the cell only and capsid-donor only controls; and (vii-xii) peak GFP object count data for S1 Fig B ΔP1 and ΔLbdcap GFP replicons, following a transfection in the first round of the assay in the presence or absence of GuHCl, and the cell only and capsid-donor only controls. (PDF) [file ppat.1012283.s001.pdf]

S1 Fig.

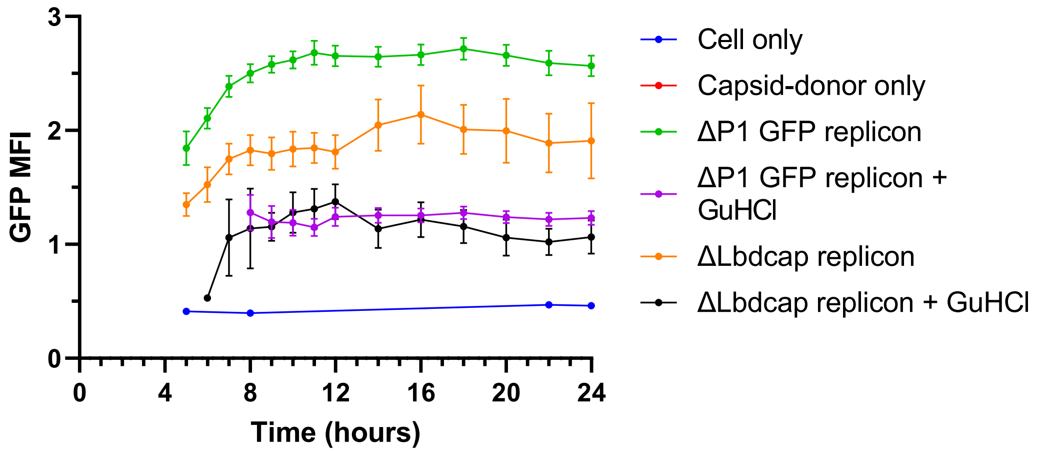

(A) Replicon GFP expression and *trans*-encapsidation efficiency in the presence of an RNA replication inhibitor. (A) The ΔP1 and ΔLbdcap GFP replicons were transfected into cells with (green and orange lines) and without (purple and black lines) the presence of GuHCl, a replication inhibitor. The green MFI was read over time using the Incucyte S3 Live cell imager to measure GFP expression levels from both replicating RNA and from input-only translation. The data shown represent the mean from triplicate wells, and the error bars represent the SEM from 12 images.

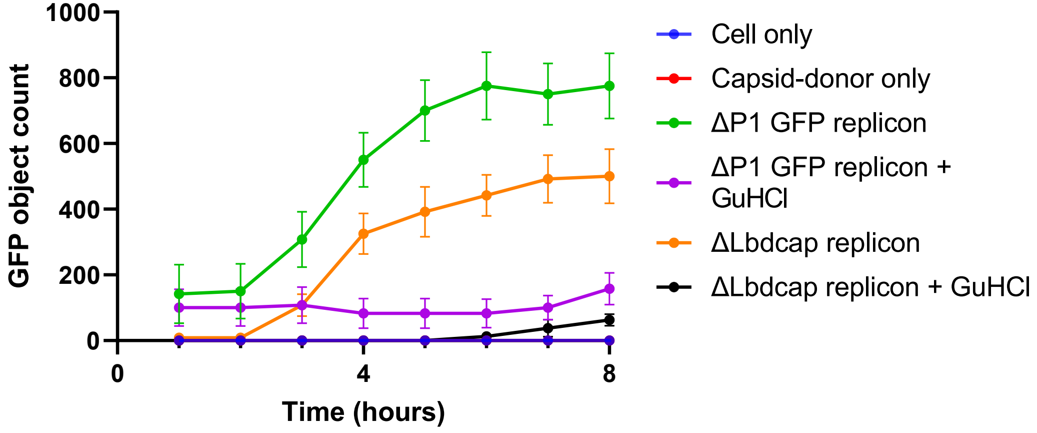

(B) Replicon GFP expression and *trans*-encapsidation efficiency in the presence of an RNA replication inhibitor. The readout of the second round of the *trans*-encapsidation assay, measured by counting the number of GFP foci, when the first round was carried out in the presence or absence of GuHCl. The data shown represent the mean from triplicate wells, and the error bars represent the SEM from 12 images.

Time (hours)	Cell only			Capsid-donor only			ΔP1 GFP replicon			ΔP1 GFP replicon + GuHCl		
	Mean	Standard deviation	Number	Mean	Standard deviation	Number	Mean	Standard deviation	Number	Mean	Standard deviation	Number
0			12			12			12			12
1			12			12			12			12
2			12			12			12			12
3			12			12			12			12
4			12			12			12			12
5	0.41156		12			12	1.843126	0.146951	12			12
6			12			12	2.106653	0.089911	12			12
7			12			12	2.385917	0.093277	12			12
8	0.396518		12			12	2.500645	0.081555	12	1.279781	0.155124	12
9			12			12	2.57879	0.073118	12	1.197268	0.139895	12
10			12			12	2.619992	0.077147	12	1.189623	0.112199	12
11			12			12	2.6806	0.104999	12	1.149447	0.074861	12
12			12			12	2.653811	0.089908	12	1.241215	0.081252	12
14			12			12	2.645872	0.087211	12	1.253802	0.063918	12
16			12			12	2.662676	0.091727	12	1.252867	0.060828	12
18			12			12	2.716064	0.094298	12	1.277061	0.056308	12
20			12			12	2.657679	0.093347	12	1.238945	0.053774	12
22	0.469949	0	12			12	2.591389	0.107735	12	1.21753	0.058147	12
24	0.460114	0	12			12	2.566976	0.08965	12	1.230093	0.060638	12

Time (hours)	ΔLbdcap replicon			ΔLbdcap replicon + GuHCl		
	Mean	Standard deviation	Number	Mean	Standard deviation	Number
0			12			12
1			12			12
2			12			12
3			12			12
4			12			12
5	1.349965	0.10235	12			12
6	1.522925	0.154202	12	0.529805	0	12
7	1.749358	0.135311	12	1.059864	0.336446	12

8	1.825585	0.132779	12	1.138027	0.34981	12
9	1.797367	0.142514	12	1.153329	0.123531	12
10	1.836798	0.151919	12	1.278752	0.177135	12
11	1.847207	0.13381	12	1.311155	0.173878	12
12	1.812147	0.147655	12	1.374098	0.151878	12
14	2.045184	0.227232	12	1.135974	0.166979	12
16	2.13858	0.254213	12	1.215394	0.152773	12
18	2.009822	0.215544	12	1.157404	0.145886	12
20	1.995676	0.28116	12	1.059501	0.158001	12
22	1.887729	0.25839	12	1.022061	0.115487	12
24	1.909353	0.330296	12	1.064146	0.145838	12

(C) Mean and Standard Error data for S1 Fig A.

Time (hours)	Cell only			Capsid-donor only			ΔP1 GFP replicon			ΔP1 GFP replicon + GuHCl		
	Mean	Standard deviation	Number	Mean	Standard deviation	Number	Mean	Standard deviation	Number	Mean	Standard deviation	Number
0	0	0	12	0	0	12	116.6667	82.41923	12	91.66667	52.884	12
1	0	0	12	0	0	12	141.6667	89.9986	12	100	56.40761	12
2	0	0	12	0	0	12	150	83.9372	12	100	56.40761	12
3	0	0	12	0	0	12	308.3333	84.79773	12	108.3333	55.67538	12
4	0	0	12	0	0	12	550	83.02975	12	83.33333	45.78165	12
5	0	0	12	0	0	12	700	92.93204	12	83.33333	45.78165	12
6	0	0	12	0	0	12	775	103.0776	12	83.33333	44.09585	12
7	0	0	12	0	0	12	750	93.33874	12	100	36.92745	12
8	0	0	12	0	0	12	775	99.33491	12	158.3333	48.39599	12
9	0	0	12	0	0	12	750	94.94815	12	225	50.93817	12
10	0	0	12	0	0	12	800	102.2475	12	291.6667	65.66482	12
11	0	0	12	0	0	12	808.3333	110.4113	12	391.6667	83.89959	12
12	0	0	12	0	0	12	833.3333	115.6885	12	416.6667	84.23752	12
14	0	0	12	0	0	12	775	95.44553	12	483.3333	102.1239	12
16	0	0	12	0	0	12	808.3333	106.9256	12	550	96.53073	12
18	0	0	12	0	0	12	766.6667	92.38697	12	558.3333	111.0953	12
20	0	0	12	0	0	12	791.6667	101.0988	12	550	101.13	12
22	25	25	12	0	0	12	808.3333	104.7785	12	575	105.9767	12
24	25	25	12	0	0	12	816.6667	109.9816	12	566.6667	109.6367	12

Time (hours)	ΔLbdcap replicon			ΔLbdcap replicon + GuHCl		
	Mean	Standard deviation	Number	Mean	Standard deviation	Number
0	16.66667	11.23666	12	0	0	12
1	8.333334	8.333333	12	0	0	12
2	8.333334	8.333333	12	0	0	12
3	108.3333	33.61622	12	0	0	12
4	325	61.69942	12	0	0	12
5	391.6667	76.33492	12	0	0	12
6	441.6667	63.31539	12	12.5	12.5	12
7	491.6667	73.29717	12	37.5	26.30521	12

8	500	82.57228	12	62.5	18.29813	12
9	491.6667	77.32099	12	200	65.46537	12
10	516.6667	80.55991	12	250	56.69467	12
11	508.3333	72.25621	12	275	70.07649	12
12	500	62.76459	12	262.5	59.57438	12
14	408.3333	64.5008	12	312.5	69.27559	12
16	358.3333	52.884	12	362.5	94.37293	12
18	325	47.87136	12	350	94.49112	12
20	283.3333	50.50253	12	287.5	85.43481	12
22	233.3333	46.60169	12	225	64.77985	12
24	175	39.16747	12	150	42.25771	12

(D) Mean and Standard Error data for S1 Fig B.

i. Cell only R1 7 hrs

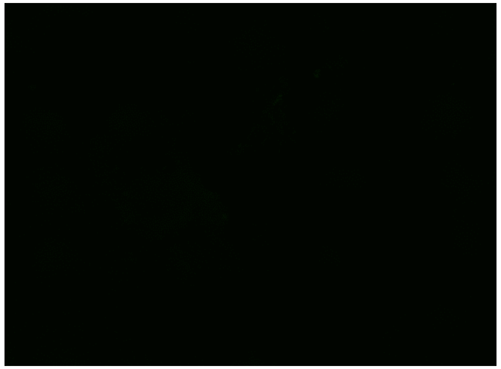

ii. Capsid-donor only R1 7 hrs

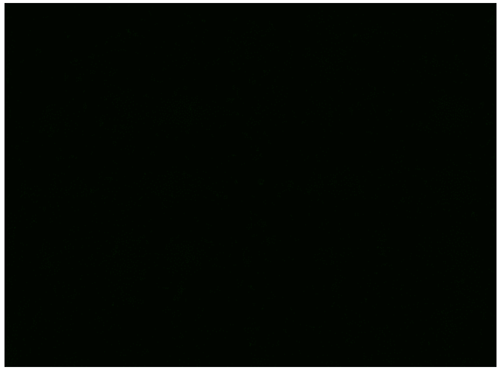

iii. ΔP1 GFP replicon R1 5 hrs

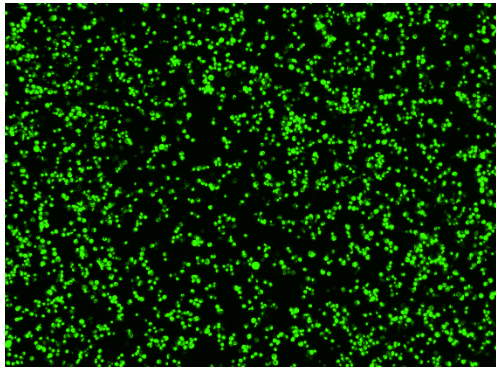

iv. ΔP1 GFP replicon R1 5 hrs + GuHCl

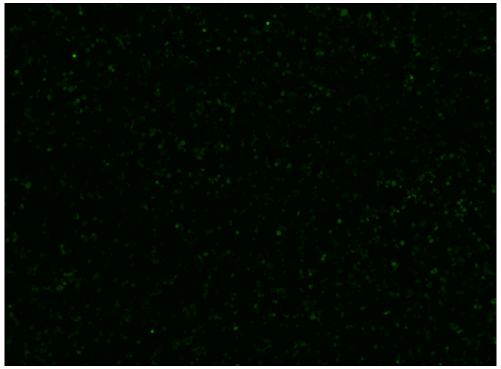

v. ΔLbdcap GFP replicon R1 7 hrs

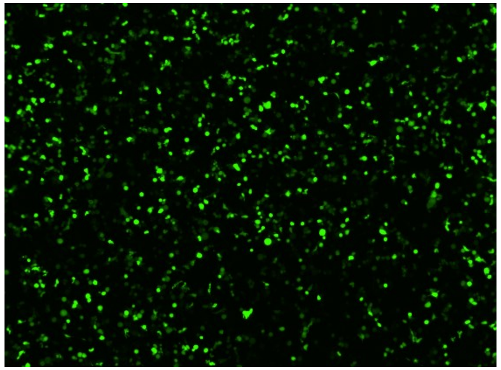

vi. ΔLbdcap GFP replicon R1 7 hrs + GuHCl

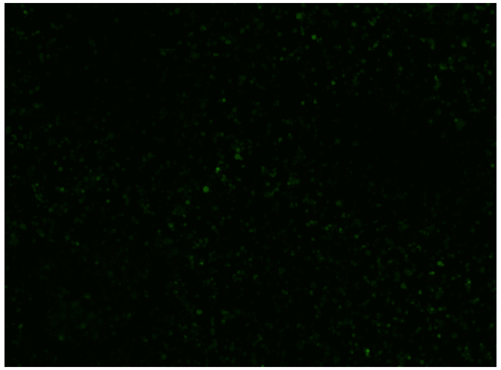

vii. Cell only R2 8 hrs

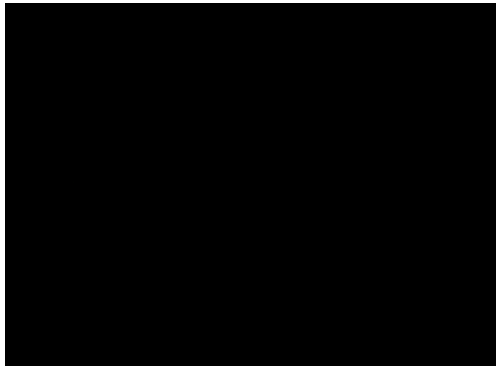

viii. Capsid-donor only R2 8 hrs

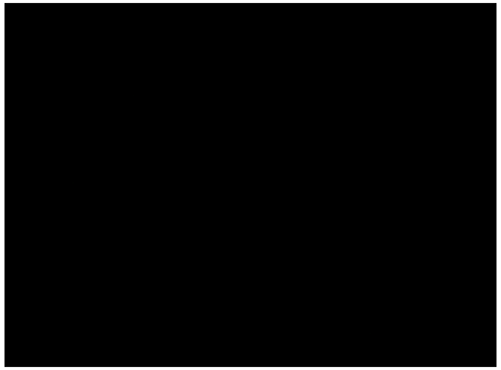

ix. ΔP1 GFP replicon R2 8 hrs

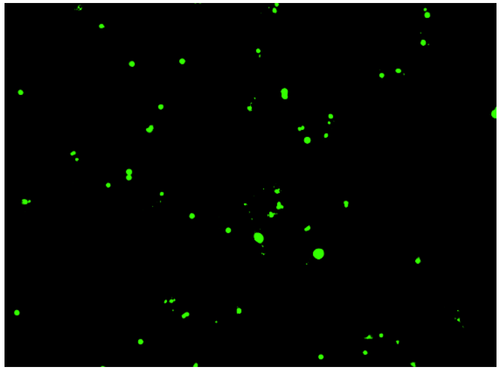

x. ΔP1 GFP replicon R2 8 hrs + GuHCl

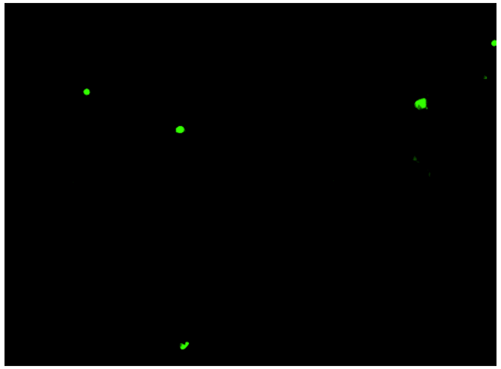

xi. ΔLbdcap GFP replicon R2 8 hrs

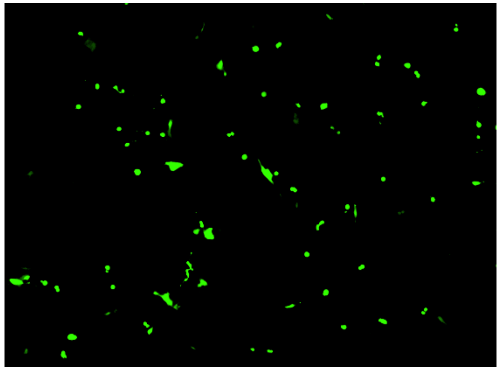

xii. ΔLbdcap GFP replicon R2 8 hrs + GuHCl

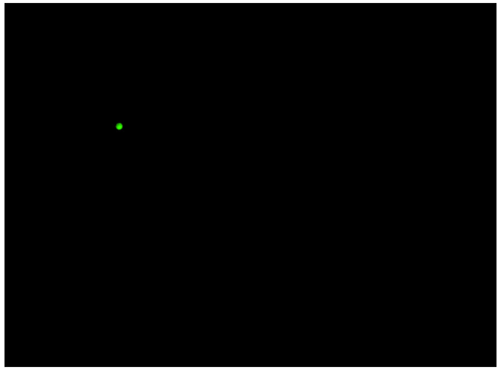

(E) Representative images analysed using the Incucyte software to obtain the: MFI data at the point of harvest for (i-vi) S1 Fig A ΔP1 and ΔLbdcap GFP replicons, in the presence and absence of GuHCl, and the cell only and capsid-donor only controls; and (vii-xii) peak GFP object count data for S1 Fig B ΔP1 and ΔLbdcap GFP replicons, following a transfection in the first round of the assay in the presence and absence of GuHCl, and the cell only and capsid-donor only controls.
