# Supplementary material for: The pseudoknot region and poly-(C) tract comprise an essential RNA packaging signal for assembly of foot-and-mouth disease virus
Source: PLoS Pathog. 2024 Dec 23;20(12):e1012283. doi: 10.1371/journal.ppat.1012283 (PMC11734982; doi:10.1371/journal.ppat.1012283)
Supplement: S2 Fig — (A) Relative stability and replication ability of transfected replicon RNA in cells. Replicon RNA transcripts were transfected into cells, washed after 15 minutes and harvested after either 15 minutes or 6 hours post-transfection (equivalent to the harvest timepoint for the trans-encapsidation assay) in the presence or absence of 3 mM GuHCl. After extracting the RNA, relative RNA yields were assessed by RT-qPCR based on the Ct values. The experiment was performed in triplicate, with the means and standard errors shown here. No significant differences were seen between the constructs with PK deletions and the equivalent sample for the wt replicon (P < 0.005), nor were marked differences seen in the presence or absence of GuHCl for each construct. The dotted line represents the cut-off point. (B) RNA abundance values, including the Mean and Standard Error data, for S1A. Values were calculated based on the Ct values (assuming 100% amplification efficiency) and multiplied by 10^12 to improve the scale for ease of interpretation. (PDF) [file ppat.1012283.s002.pdf]

S2 Fig.

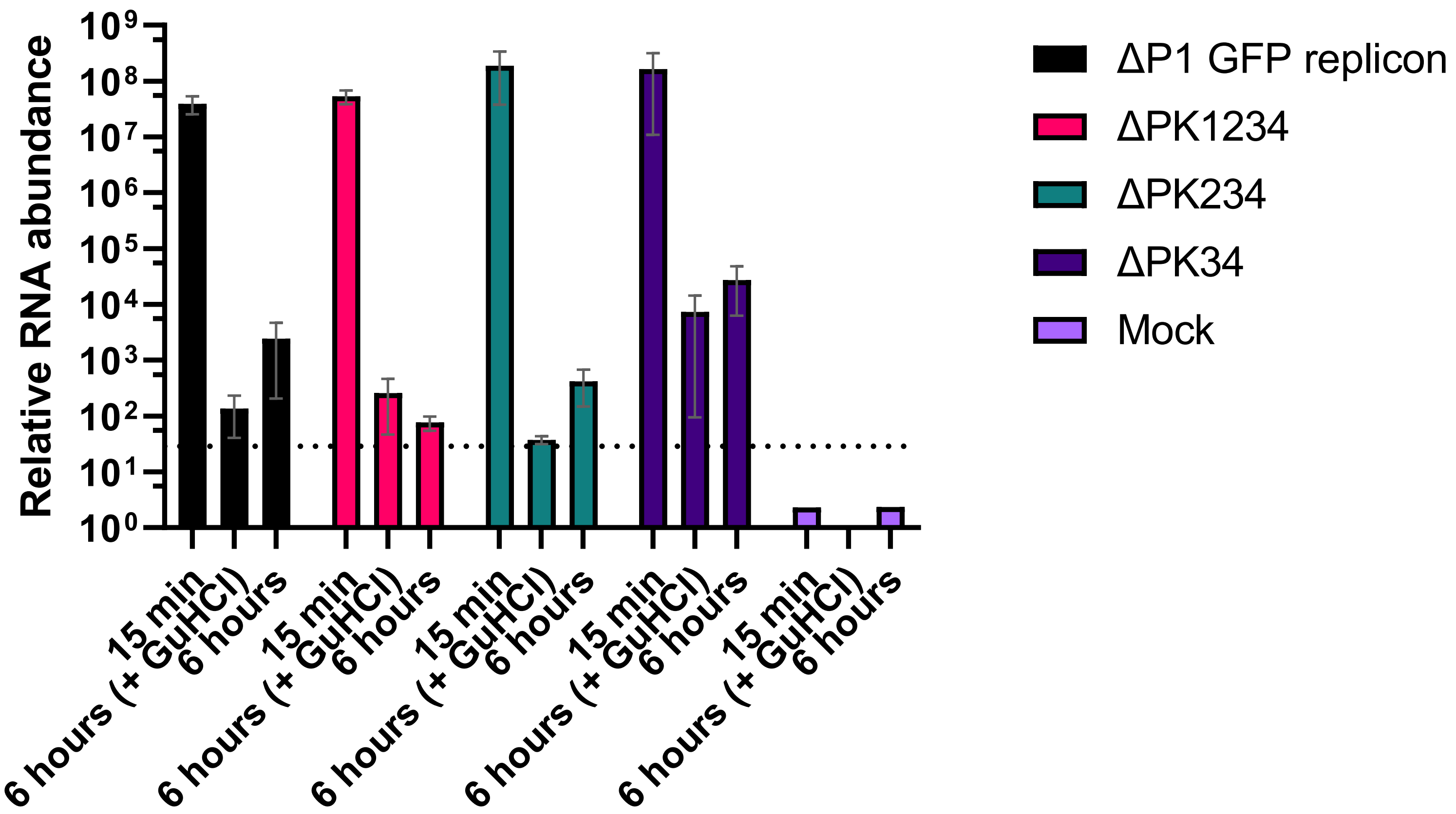

(A) Relative stability and replication ability of transfected replicon RNA in cells. Replicon RNA transcripts were transfected into cells, washed after 15 minutes and harvested after either 15 minutes or 6 hours post-transfection (equivalent to the harvest timepoint for the trans-encapsidation assay) in the presence or absence of 3 mM GuHCl. After extracting the RNA, relative RNA yields were assessed by RT-qPCR based on the Ct values. The experiment was performed in triplicate, with the means and standard errors shown here. No significant differences were seen between the constructs with PK deletions and the equivalent sample for the wt replicon ($P < 0.005$), nor were marked differences seen in the presence or absence of GuHCl for each construct. The dotted line represents the cut-off point.

	ΔP1 GFP replicon			Mean	Standard deviation	ΔPK1234			Mean	Standard deviation
15 min	49233506	49920780	19448252	39534179	17398317	58000000	33361004	68193795	53184933	17908643
6 hours + GuHCl	260.9183	40.8947	110.487	137.4333	112.4597	271.3564	25.2975	471.1976	255.9505	223.3489
6 hours	5300.5618	1800.166	206.9498	2435.892	2605.635	80.5472	98.9712	50.3739	76.63077	24.53423

	ΔPK234			Mean	Standard deviation	ΔPK34			Mean	Standard deviation
15 min	87521683	4.05E+08	77793007	1.9E+08	1.86E+08	98467012	18527144	3.78E+08	1.65E+08	1.89E+08
6 hours + GuHCl	36.2539	32.4939	43.3	37.34927	5.485692	6300.258	1010.369	14572.94	7294.523	6835.735
6 hours	452.1547	142.4582	652.1547	415.5892	256.8081	54847.46	4554.69	21968.32	27123.49	25539.63

15 min	Mock			Mean	Standard deviation
6 hours + GuHCl		2.318412		2.318412	
6 hours					
			2.350776	2.350776	

(B) RNA abundance values, including the Mean and Standard Error data, for S1 Fig A. Values were calculated based on the ct values (assuming 100% polymerase efficiency) and multiplied by 10^12 to improve the scale for ease of interpretation.
