# Supplementary material for: The pseudoknot region and poly-(C) tract comprise an essential RNA packaging signal for assembly of foot-and-mouth disease virus
Source: PLoS Pathog. 2024 Dec 23;20(12):e1012283. doi: 10.1371/journal.ppat.1012283 (PMC11734982; doi:10.1371/journal.ppat.1012283)
Supplement: S3 Fig — (A-C) Mean and Standard Error data for Fig 3A–3C respectively. (D) Representative images analysed using the Incucyte software to obtain the: GFP object count and MFI data at the point of harvest for Fig 3A and 3B (i) ΔP1 GFP replicon, (ii) ΔLbdcap GFP replicon, (iii) Cell only and (iv) Capsid-donor only; and peak GFP object count data for Fig 3C (v) ΔP1 GFP replicon, (vi) ΔLbdcap GFP replicon and (vii) transfected replicon only. (PDF) [file ppat.1012283.s003.pdf]

S3 Fig.

Time (hours)	Cell only			Capsid-donor only			ΔP1 GFP replicon			ΔLbdcap GFP replicon		
	Mean	Standard deviation	Number	Mean	Standard deviation	Number	Mean	Standard deviation	Number	Mean	Standard deviation	Number
2	3.139186	0.410881	5	2.507007	0.083217	5	2.623359	0.127814	5	3.026958	0.31351	5
3	5.361324	1.721044	5	2.687055	0.499555	5	4.713941	0.189188	5	2.828398	0.02844	5
4	4.273342	0.909821	5	3.384033	0	5	8.025169	0.398857	5	3.511057	0.099845	5
5	4.146493	0.94017	5	3.055977	0	5	11.25586	0.442314	5	6.079836	0.294942	5
6	4.064102	0.773885	5	3.254552	0	5	14.03183	0.463308	5	9.055323	0.440406	5
7	4.498331	1.218768	5	3.382586	0	5	15.64889	0.431041	5	11.11747	0.534672	5
8	4.689428	1.228469	5	3.050157	0.238802	5	16.39816	0.446866	5	12.48793	0.550716	5
9	3.885963	0.679993	5	2.878397	0.266811	5	16.69841	0.429211	5	13.39051	0.605632	5
10	4.795829	1.191767	5	3.062222	0.227884	5	16.74704	0.448059	5	13.94794	0.613348	5
11	4.056757	0.622248	5	3.037176	0.075309	5	16.58614	0.409515	5	14.24082	0.657815	5
12	3.93114	0.396589	5	3.113808	0.010429	5	16.45333	0.435592	5	14.51339	0.657857	5
13	3.987159	0.689319	5	3.106594	0.049614	5	16.20439	0.436796	5	14.80585	0.655586	5
14	4.598684	1.272094	5	3.062587	0.082639	5	15.98841	0.46804	5	15.06829	0.652124	5
15	4.560997	1.178309	5	3.316758	0.061981	5	15.65489	0.47917	5	15.39767	0.68465	5
16	4.699455	1.217311	5	3.318465	0.188657	5	15.3529	0.533661	5	15.68555	0.710967	5
17	5.219799	1.501468	5	3.018868	0.170413	5	14.94244	0.54812	5	15.91704	0.728692	5
18	3.960554	0.489071	5	3.338043	0.03669	5	14.6246	0.581053	5	16.17183	0.761933	5
19	4.468773	1.17038	5	3.447309	0.270639	5	14.22456	0.626797	5	16.48728	0.786922	5
20	4.751427	1.133526	5	3.406708	0.154037	5	13.79093	0.652588	5	16.52781	0.797494	5
21	4.709819	1.113033	5	3.220912	0.014418	5	13.45978	0.675934	5	16.49205	0.832148	5
22	3.932629	0.35582	5	3.200104	0.25075	5	13.07944	0.718941	5	16.49426	0.822793	5
23	4.802645	1.091189	5	3.3149	0.117639	5	12.67056	0.743347	5	16.42999	0.84015	5
24	4.774482	1.066981	5	3.239878	0.174347	5	12.32549	0.773977	5	16.36174	0.826084	5

(A) Mean and Standard Error data for Fig 3A.

Time (hours)	ΔP1 GFP replicon			LLdcap GFP replicon			Capsid-donor only only		
	Mean	Standard deviation	Number	Mean	Standard deviation	Number	Mean	Standard deviation	Number
2	78.6	53.86985	5	499.6	99.9928	5	218.4	140.6818	5
3	68545.8	7708.711	5	6060.4	753.0255	5	226.2	155.5077	5
4	267555.4	11707.46	5	78804	6453.062	5	13	13	5
5	295633.2	15014.23	5	186652.6	9286.452	5	7.8	7.8	5
6	289343	15362.68	5	244556.6	10192.32	5	10.4	10.4	5
7	285083	15205.82	5	263079.4	11058.2	5	10.4	7.580237	5
8	281454.6	15137.49	5	264682.6	11586.66	5	10.4	10.4	5
9	280259.2	15046.77	5	259970	11450.6	5	10.4	10.4	5
10	282399.8	16055.52	5	257602.8	11580.94	5	13	10.06976	5
11	282115.6	16725.65	5	250009.4	10845.88	5	7.8	7.8	5
12	280980.8	17053.68	5	241441.6	10400.26	5	7.8	7.8	5
13	279217	17058.09	5	235167.2	9806.966	5	7.8	7.8	5
14	277497.2	16564.13	5	227734.6	10025.68	5	13	10.06976	5
15	275204.2	17067.46	5	221294.4	9483.413	5	10.4	7.580237	5
16	274179.8	18224.23	5	212853.2	8936.033	5	7.8	5.2	5
17	272879	18052.83	5	206599.8	8467.567	5	10.4	7.580237	5
18	274453.6	17676.89	5	196936.8	8301.098	5	7.8	5.2	5
19	274761.8	18029.95	5	190859.6	7357.665	5	5.2	5.2	5
20	277623.8	19236.44	5	185088.4	7857.958	5	5.2	5.2	5
21	279000.8	20647.95	5	179651.4	7745.507	5	5.2	5.2	5
22	283118.8	20852.12	5	173400.8	7650.699	5	5.2	5.2	5
23	283829.6	21920.34	5	169572.4	7430.81	5	7.8	7.8	5
24	287589.6	22403.54	5	163443.2	7287.003	5	5.2	5.2	5

(B) Mean and Standard Error data for Fig 3B.

Time (hours)	ΔP1 GFP replicon Mean	Standard deviation	Number	LLdcap GFP replicon Mean	Standard deviation	Number	Transfected replicon only Mean	Standard deviation	Number
0	13	3.919647	12	0	0	12	0	0	12
1	6.5	3.394514	12	0	0	12	0	0	12
2	4.333333	2.921533	12	0	0	12	0	0	12
3	63	13.06221	12	6.5	4.665314	12	0	0	12
4	132.75	23.58001	12	78.33334	23.69386	12	0	0	12
5	241.8333	36.7827	12	178.6667	39.07013	12	0	0	12
6	285.6667	39.77481	12	270.3333	48.3222	12	0	0	12
7	309.6667	36.5862	12	320.5833	52.38631	12	0	0	12
8	311.75	39.40219	12	392.5833	62.18941	12	0	0	12
9	340.25	36.52917	12	434.0833	60.96913	12	0	0	12
10	348.9167	41.57131	12	440.8333	63.03593	12	0	0	12
11	364.0833	44.27676	12	427.5833	61.95129	12	0	0	12
12	361.9167	43.97321	12	458.1667	60.89644	12	0	0	12
13	368.75	40.33367	12	462.5	58.27956	12	0	0	12
14	351.25	38.89927	12	462.4167	59.2572	12	0	0	12
15	357.6667	37.39234	12	447.1667	50.8679	12	0	0	12
16	355.5833	32.6082	12	434.1667	43.80152	12	0	0	12

(C) Mean and Standard Error data for Fig 3C.

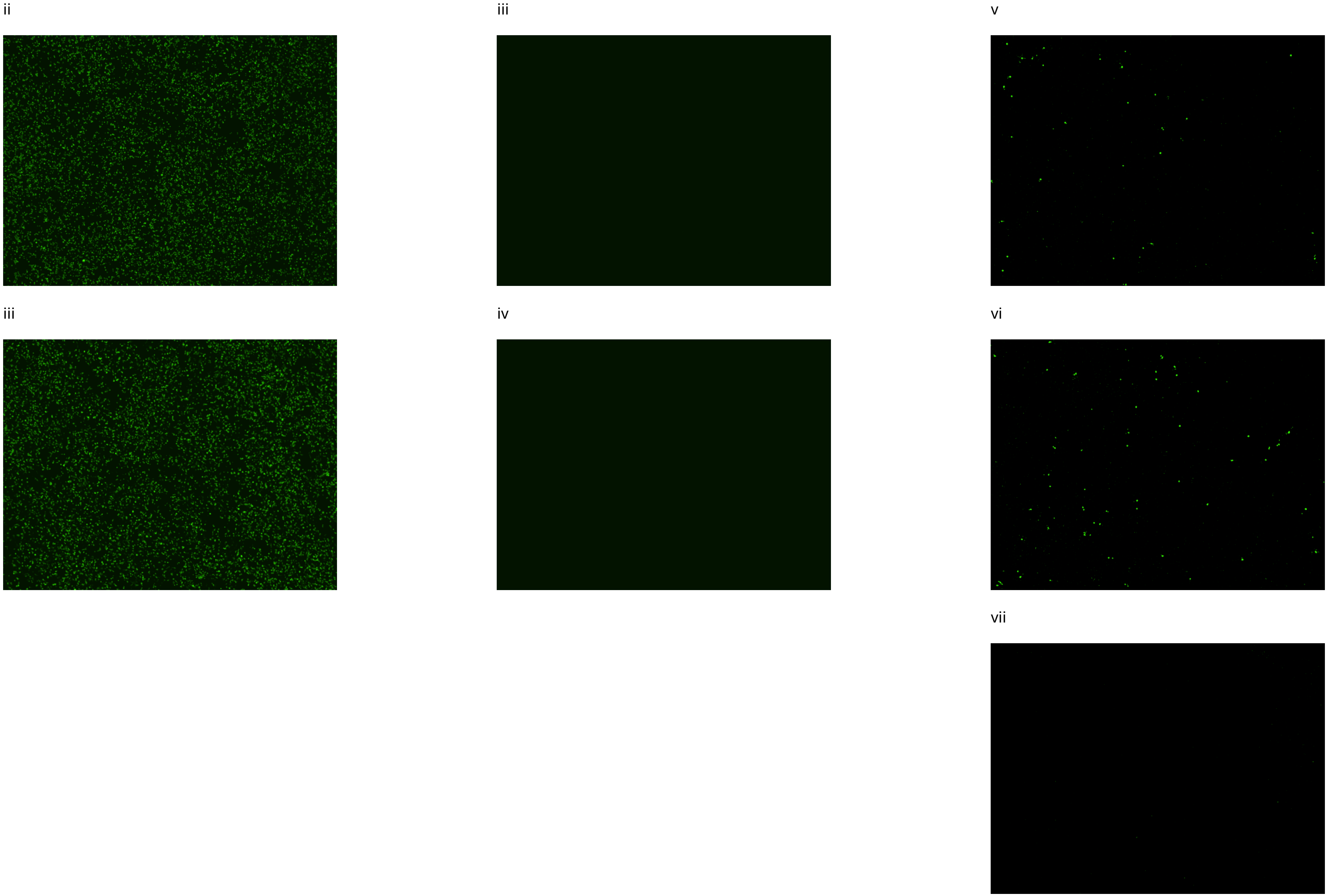

(D) Representative images analysed using the Incucyte software to obtain the: GFP object count and MFI data at the point of harvest for Fig 3A and Fig 3B (i) ΔP1 GFP replicon, (ii) ΔLbdcap GFP replicon, (iii) Cell only and (iv) Capsid-donor only; and peak GFP object count data for Fig 3C (v) ΔP1 GFP replicon, (vi) ΔLbdcap GFP replicon and (vii) transfected replicon only.
