# Supplementary material for: The pseudoknot region and poly-(C) tract comprise an essential RNA packaging signal for assembly of foot-and-mouth disease virus
Source: PLoS Pathog. 2024 Dec 23;20(12):e1012283. doi: 10.1371/journal.ppat.1012283 (PMC11734982; doi:10.1371/journal.ppat.1012283)
Supplement: S4 Fig — (A-E) Mean and Standard Error data for Fig 5A–5E respectively. (F) Representative images analysed using the Incucyte software to obtain the: (i-iv) GFP object count and MFI data at the point of harvest for Fig 5A and 5B ΔP1 C11, C29 and C35 GFP replicons and cell only control; (v-viii) peak GFP object count data for Fig 5C ΔP1 C11, C29 and C35 GFP replicons and transfected replicon only control; (ix-xx) monolayer confluency to measure CPE for the C11, C29 and C35 ICs and cell only control at 0, 12 and 24 hrs for Fig 5D; (xxi-xxiv) and peak GFP object count data for Fig 5C ΔP1 C11, C29 and C35 GFP replicons and transfected replicon only control for Fig 5E. (PDF) [file ppat.1012283.s004.pdf]

**S4 Fig.**

| C11 replicon | | | C29 replicon | | | C35 (*wt*) replicon | | | Cell only | | |
|---|---|---|---|---|---|---|---|---|---|---|---|
| Mean | Standard deviation | Number | Mean | Standard deviation | Number | Mean | Standard deviation | Number | Mean | Standard deviation | Number |
| 14190 | 870.2336 | 5 | 14370.8 | 720.4975 | 5 | 12868.6 | 346.9301 | 5 | 1.6 | 0.979796 | 5 |

**(A) Mean and Standard Error data for Fig 5A.**

| C11 replicon | | | C29 replicon | | | C35 (*wt*) replicon | | | Cell only | | |
|---|---|---|---|---|---|---|---|---|---|---|---|
| Mean | Standard deviation | Number | Mean | Standard deviation | Number | Mean | Standard deviation | Number | Mean | Standard deviation | Number |
| 8.510814 | 0.267824 | 5 | 8.222754 | 0.237869 | 5 | 8.633967 | 0.222126 | 5 | 2.65014 | 0.168038 | 5 |

**(B) Mean and Standard Error data for Fig 5B.**

| C11 replicon | | | C29 replicon | | | C35 (*wt*) replicon | | | Transfected replicon only | | |
|---|---|---|---|---|---|---|---|---|---|---|---|
| Mean | Standard deviation | Number | Mean | Standard deviation | Number | Mean | Standard deviation | Number | Mean | Standard deviation | Number |
| 1509.867 | 54.36874 | 12 | 4065.6 | 167.4673 | 12 | 5699.867 | 68.87289 | 12 | 0 | 0 | 12 |

**(C) Mean and Standard Error data for Fig 5C.**

| Time (hours) | C11 | | | C29 | | | C35 wt | | | Cell only | | |
|---|---|---|---|---|---|---|---|---|---|---|---|---|
| | Mean | Standard deviation | Number | Mean | Standard deviation | Number | Mean | Standard deviation | Number | Mean | Standard deviation | Number |
| 1 | 76.01188 | 1.936223 | 12 | 73.8786 | 2.686878 | 12 | 76.64013 | 1.686143 | 12 | 67.03728 | 2.939706 | 12 |
| 2 | 74.03135 | 2.00173 | 12 | 70.31281 | 2.752347 | 12 | 72.98004 | 1.770204 | 12 | 64.49027 | 3.094554 | 12 |
| 3 | 71.05717 | 2.116707 | 12 | 66.67892 | 2.95488 | 12 | 68.85272 | 1.950272 | 12 | 66.8233 | 3.135401 | 12 |
| 4 | 66.31221 | 2.232322 | 12 | 60.9649 | 2.939769 | 12 | 62.01572 | 1.98007 | 12 | 69.39036 | 3.173708 | 12 |
| 5 | 62.38395 | 2.269351 | 12 | 56.49059 | 2.943405 | 12 | 57.29946 | 2.051244 | 12 | 72.34708 | 3.060384 | 12 |
| 6 | 60.02111 | 2.261221 | 12 | 54.87959 | 2.957891 | 12 | 55.43456 | 2.087832 | 12 | 74.70209 | 2.954133 | 12 |
| 7 | 59.12511 | 2.42684 | 12 | 53.16691 | 3.009358 | 12 | 53.83358 | 2.134662 | 12 | 76.84074 | 2.912533 | 12 |
| 8 | 58.18447 | 2.401314 | 12 | 52.56033 | 3.020677 | 12 | 53.27971 | 2.14607 | 12 | 79.10652 | 2.757398 | 12 |
| 9 | 57.03917 | 2.425047 | 12 | 51.911 | 3.078288 | 12 | 51.98738 | 2.189282 | 12 | 80.60139 | 2.731203 | 12 |
| 10 | 56.12304 | 2.54313 | 12 | 50.78182 | 3.089787 | 12 | 50.85735 | 2.232002 | 12 | 82.6801 | 2.601442 | 12 |
| 11 | 54.29704 | 2.599039 | 12 | 49.55231 | 3.101311 | 12 | 49.61667 | 2.260693 | 12 | 83.75435 | 2.518761 | 12 |
| 12 | 53.01635 | 2.623885 | 12 | 48.20422 | 3.052552 | 12 | 47.83722 | 2.256072 | 12 | 85.11629 | 2.386306 | 12 |
| 13 | 50.75188 | 2.671861 | 12 | 46.82286 | 3.08438 | 12 | 46.59505 | 2.304051 | 12 | 86.51563 | 2.215525 | 12 |
| 14 | 48.62089 | 2.666784 | 12 | 45.21727 | 3.075516 | 12 | 44.90852 | 2.267431 | 12 | 87.70415 | 2.096665 | 12 |
| 15 | 46.41755 | 2.662407 | 12 | 43.38873 | 3.054435 | 12 | 43.5704 | 2.283072 | 12 | 88.9484 | 2.031905 | 12 |
| 16 | 44.55793 | 2.639186 | 12 | 42.04097 | 2.999765 | 12 | 41.62911 | 2.203618 | 12 | 90.0591 | 1.926118 | 12 |
| 17 | 42.90059 | 2.58088 | 12 | 40.77118 | 2.954733 | 12 | 40.38287 | 2.170831 | 12 | 91.24268 | 1.73154 | 12 |
| 18 | 41.35562 | 2.570462 | 12 | 39.29828 | 2.925504 | 12 | 38.94978 | 2.108448 | 12 | 92.28115 | 1.664407 | 12 |
| 19 | 39.50394 | 2.483945 | 12 | 37.43855 | 2.8055 | 12 | 37.30101 | 2.038116 | 12 | 92.90946 | 1.589314 | 12 |
| 20 | 37.89088 | 2.464298 | 12 | 36.15773 | 2.742612 | 12 | 36.45249 | 2.009188 | 12 | 93.72169 | 1.419137 | 12 |
| 21 | 36.43443 | 2.379429 | 12 | 34.97275 | 2.65866 | 12 | 34.96673 | 1.902877 | 12 | 94.60029 | 1.341797 | 12 |
| 22 | 35.1699 | 2.320957 | 12 | 33.92819 | 2.591941 | 12 | 33.85595 | 1.861968 | 12 | 95.39658 | 1.156934 | 12 |
| 23 | 33.95793 | 2.221381 | 12 | 32.57011 | 2.484305 | 12 | 32.58764 | 1.773461 | 12 | 96.17361 | 0.977715 | 12 |
| 24 | 33.11858 | 2.162179 | 12 | 31.48422 | 2.346859 | 12 | 31.56011 | 1.657095 | 12 | 96.44491 | 0.963788 | 12 |
| 25 | 32.09174 | 2.057446 | 12 | 30.54689 | 2.233468 | 12 | 30.6531 | 1.55309 | 12 | 97.0253 | 0.795517 | 12 |
| 26 | 31.12047 | 1.95105 | 12 | 29.63675 | 2.145389 | 12 | 29.67377 | 1.506664 | 12 | 97.21975 | 0.833155 | 12 |
| 27 | 30.30708 | 1.854347 | 12 | 28.82895 | 2.026326 | 12 | 28.8793 | 1.420444 | 12 | 97.64544 | 0.698931 | 12 |
| 28 | 29.68791 | 1.791325 | 12 | 28.23653 | 1.927735 | 12 | 28.21153 | 1.309049 | 12 | 97.93162 | 0.604984 | 12 |
| 29 | 28.77966 | 1.694864 | 12 | 27.7371 | 1.866418 | 12 | 27.72024 | 1.256246 | 12 | 98.2499 | 0.574671 | 12 |
| 30 | 28.40216 | 1.612606 | 12 | 27.19628 | 1.745991 | 12 | 27.07972 | 1.192533 | 12 | 98.47694 | 0.494952 | 12 |
| 31 | 27.64426 | 1.514709 | 12 | 26.85229 | 1.678204 | 12 | 26.75451 | 1.171004 | 12 | 98.71149 | 0.441073 | 12 |
| 32 | 27.27173 | 1.442247 | 12 | 26.47302 | 1.615997 | 12 | 26.36431 | 1.127824 | 12 | 98.79081 | 0.404028 | 12 |

| | | | | | | | | | | | | |
|---|---|---|---|---|---|---|---|---|---|---|---|---|
| 33 | 26.9504 | 1.378074 | 12 | 26.05583 | 1.558985 | 12 | 25.94854 | 1.115464 | 12 | 98.85943 | 0.403848 | 12 |
| 34 | 26.76893 | 1.346669 | 12 | 25.86163 | 1.475201 | 12 | 25.67958 | 1.064759 | 12 | 99.07308 | 0.306096 | 12 |
| 35 | 26.32174 | 1.297922 | 12 | 25.57276 | 1.435809 | 12 | 25.42517 | 1.034622 | 12 | 99.1173 | 0.302833 | 12 |
| 36 | 26.01625 | 1.285527 | 12 | 25.26457 | 1.392145 | 12 | 25.26195 | 1.0138 | 12 | 99.14165 | 0.316549 | 12 |
| 37 | 25.88655 | 1.225242 | 12 | 25.06844 | 1.351822 | 12 | 24.83419 | 0.977664 | 12 | 99.32642 | 0.241234 | 12 |
| 38 | 25.50055 | 1.212125 | 12 | 25.17107 | 1.315339 | 12 | 25.0677 | 0.97921 | 12 | 99.36541 | 0.250208 | 12 |
| 39 | 25.44446 | 1.161154 | 12 | 24.92566 | 1.304181 | 12 | 24.89236 | 0.959803 | 12 | 99.46811 | 0.202209 | 12 |
| 40 | 25.45715 | 1.14588 | 12 | 24.75725 | 1.287106 | 12 | 24.7501 | 0.933077 | 12 | 99.63453 | 0.128863 | 12 |
| 41 | 25.30148 | 1.116323 | 12 | 24.74032 | 1.265067 | 12 | 24.63729 | 0.916029 | 12 | 99.64331 | 0.130231 | 12 |
| 42 | 25.11969 | 1.116315 | 12 | 24.73979 | 1.249004 | 12 | 24.40036 | 0.894974 | 12 | 99.6739 | 0.112079 | 12 |
| 43 | 24.98446 | 1.093658 | 12 | 24.55361 | 1.223967 | 12 | 24.13487 | 0.896869 | 12 | 99.70916 | 0.097589 | 12 |
| 44 | 24.78509 | 1.086614 | 12 | 24.50047 | 1.227961 | 12 | 24.37385 | 0.904625 | 12 | 99.67126 | 0.112073 | 12 |
| 45 | 24.75192 | 1.075989 | 12 | 24.44289 | 1.231157 | 12 | 24.20481 | 0.886106 | 12 | 99.74044 | 0.083011 | 12 |
| 46 | 24.87923 | 1.05901 | 12 | 24.35174 | 1.184854 | 12 | 24.0671 | 0.868468 | 12 | 99.7625 | 0.059447 | 12 |
| 47 | 24.66982 | 1.048592 | 12 | 24.3201 | 1.210796 | 12 | 24.09699 | 0.883642 | 12 | 99.72234 | 0.105736 | 12 |
| 48 | 24.55009 | 1.030494 | 12 | 24.32726 | 1.216035 | 12 | 24.18268 | 0.886597 | 12 | 99.76791 | 0.083425 | 12 |

**(D) Mean and Standard Deviation data for Fig 5D.**

| C11 helper | | | C29 helper | | | C35 (*wt*) helper | | | Transfected replicon only | | |
|---|---|---|---|---|---|---|---|---|---|---|---|
| Mean | Standard deviation | Number | Mean | Standard deviation | Number | Mean | Standard deviation | Number | Mean | Standard deviation | Number |
| 7514.417 | 257.7903 | 12 | 3664.083 | 196.4109 | 12 | 3327.833 | 144.1393 | 12 | 2.166667 | 2.166667 | 12 |

**(E) Mean and Standard Error data for Fig 5E.**

i. Fig 5A/B C11 replicon R1 5 hrs

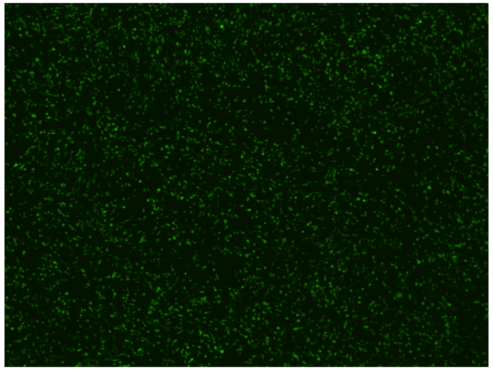

ii. Fig 5A/B C29 replicon R1 5 hrs

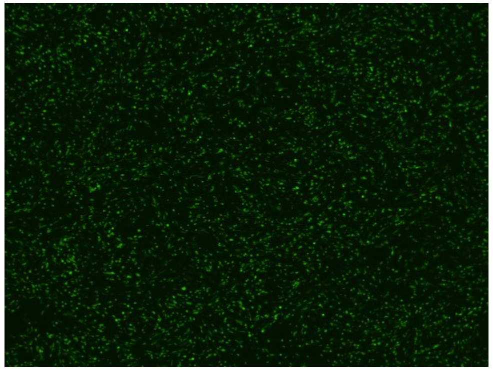

iii. Fig 5A/B C35 *wt* replicon R1 5 hrs

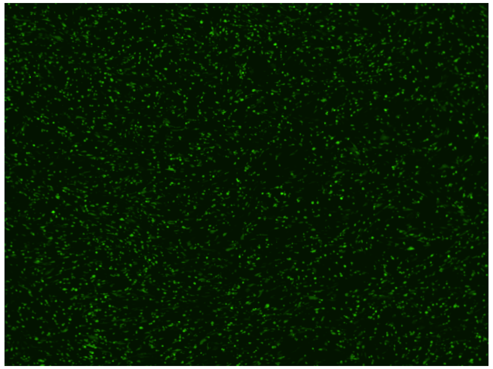

iv. Fig 5A/B cell only R1 5 hrs

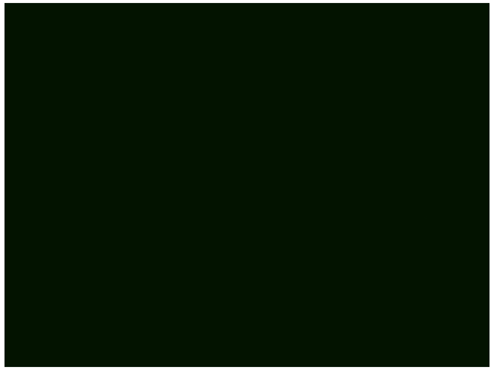

v. Fig 5C C11 replicon R2 8 hrs

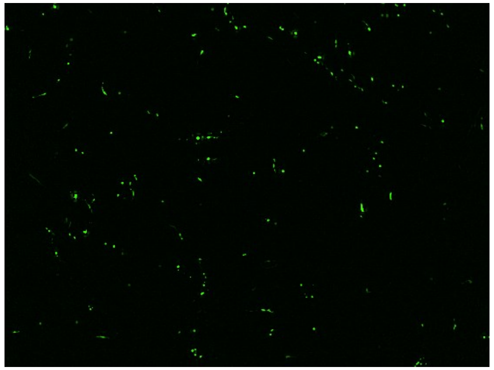

vi. Fig 5C C29 replicon R2 8 hrs

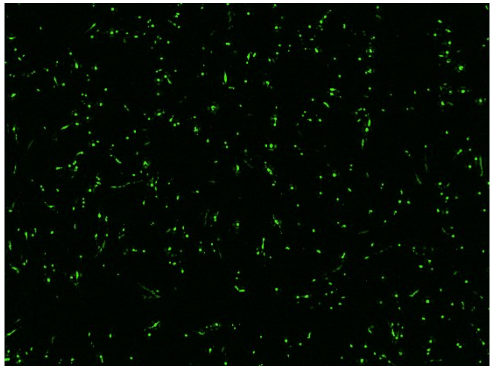

vii. Fig 5C C35 *wt* replicon R2 8 hrs

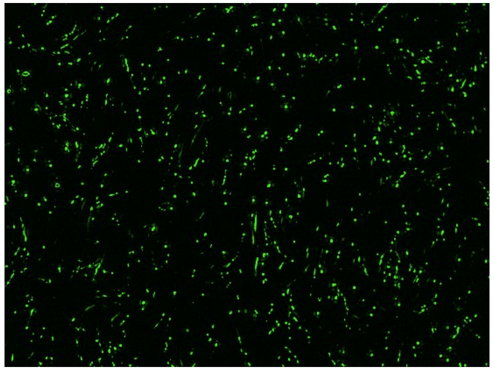

viii. Fig 5C transfected replicon only R2 8 hrs

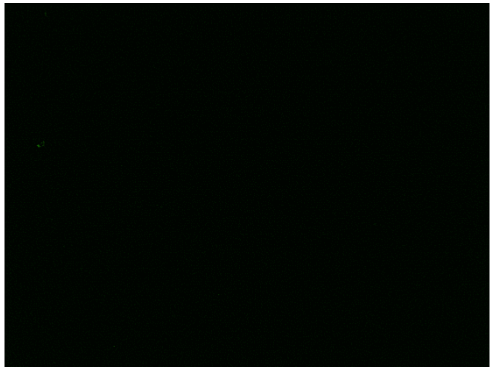

ix. Fig 5D C11 0 hrs

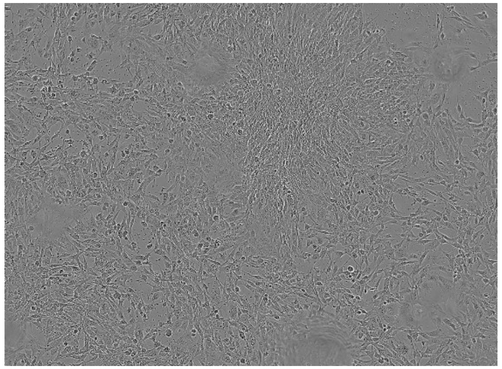

x. Fig 5D C11 12 hrs

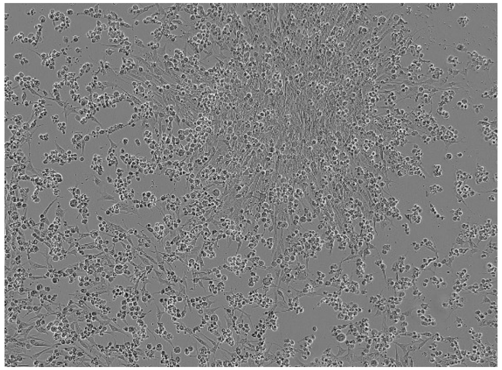

xi. Fig 5D C11 24 hrs

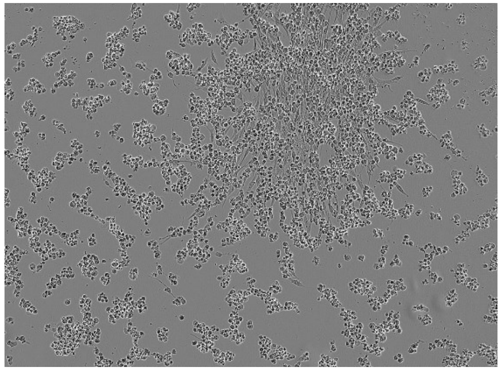

xii. Fig 5D C29 0 hrs

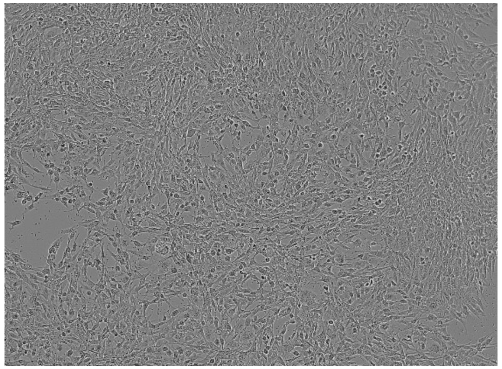

xiii. Fig 5D C29 12 hrs

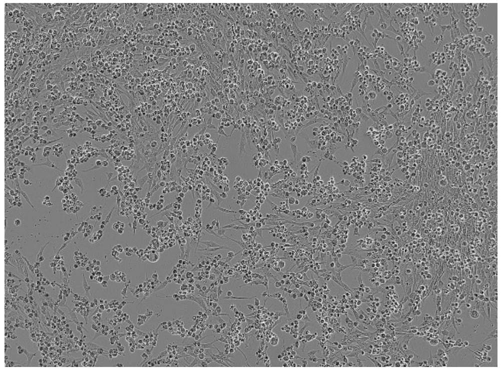

xiv. Fig 5D C29 24 hrs

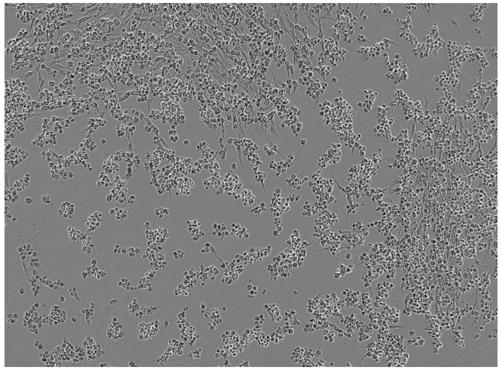

xv. Fig 5D C35 *wt* 0 hrs

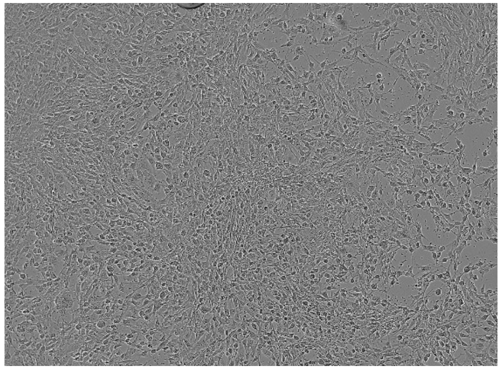

xvi. Fig 5D C35 *wt* 12 hrs

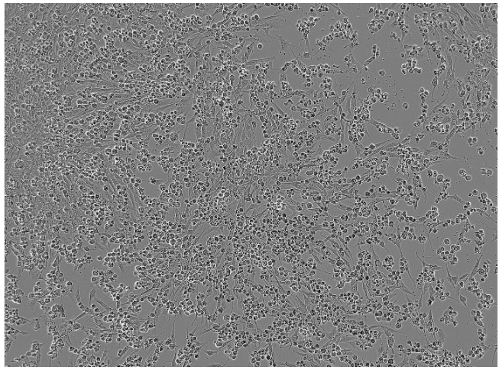

xvii. Fig 5D C35 *wt* 24 hrs

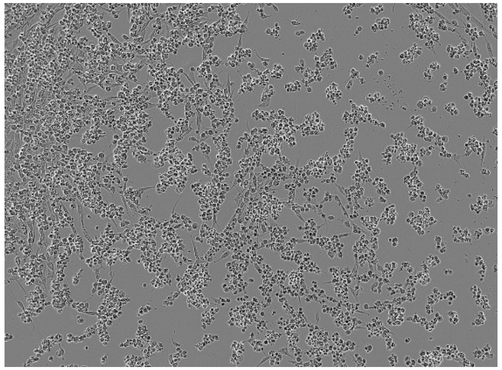

xviii. Fig 5D Cell only 0 hrs

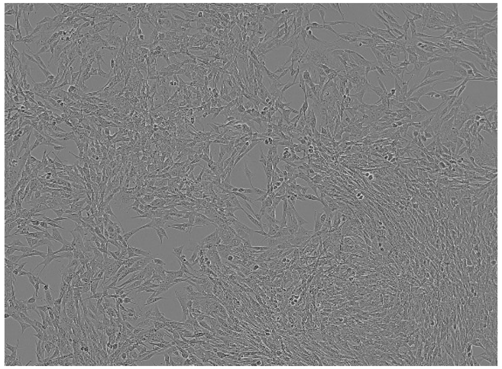

xix. Fig 5D Cell only 12 hrs

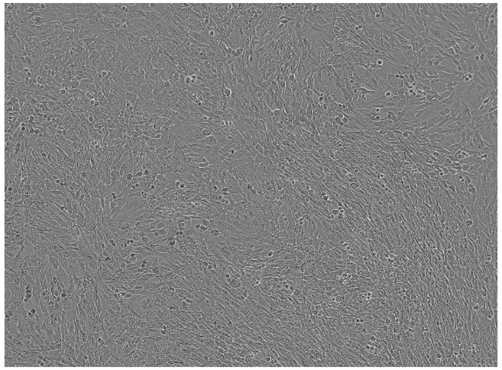

xx. Fig 5D Cell only 24 hrs

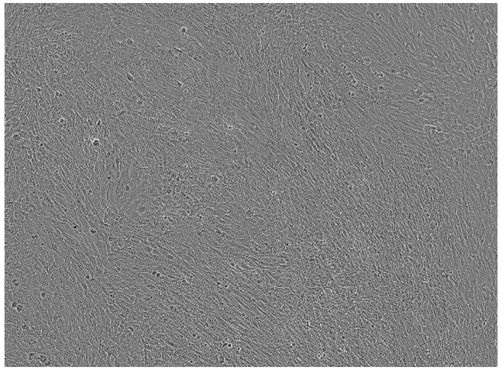

xxi. Fig 5E C11 capsid-donor R2 8 hrs

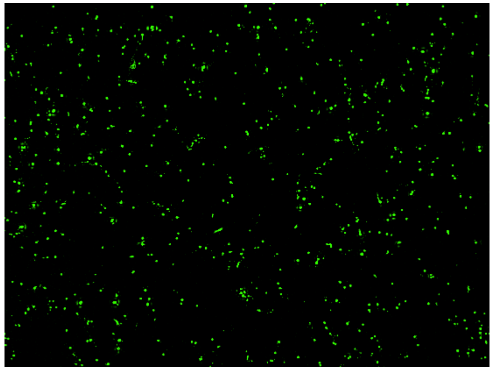

xxii. Fig 5E C29 capsid-donor R2 8 hrs

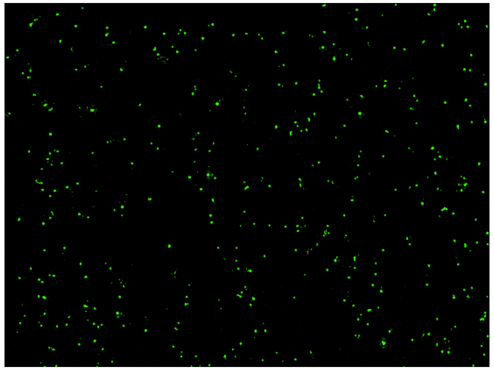

xxiii. Fig 5E C35 capsid-donor R2 8 hrs

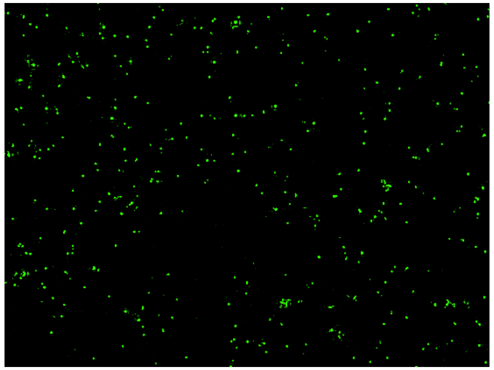

xxiv. Fig 5E transfected replicon only R2 8 hrs

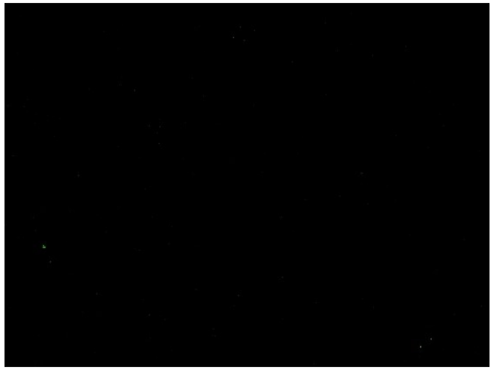

**(F) Representative images analysed using the Incucyte software** to obtain the: (i-iv) GFP object count and MFI data at the point of harvest for Fig 5A and Fig 5B ΔP1 C11, C29 and C35 GFP replicons and cell only control; (v-viii) peak GFP object count data for Fig 5C ΔP1 C11, C29 and C35 GFP replicons and transfected replicon only control; (ix-xx) monolayer confluency to measure CPE for the C11, C29 and C35 ICs and cell only control at 0, 12 and 24 hrs for Fig 5D; (xxi-xxiv) and peak GFP object count data for Fig 5C ΔP1 C11, C29 and C35 GFP replicons and transfected replicon only control for Fig 5E.
