# Supplementary material for: The pseudoknot region and poly-(C) tract comprise an essential RNA packaging signal for assembly of foot-and-mouth disease virus
Source: PLoS Pathog. 2024 Dec 23;20(12):e1012283. doi: 10.1371/journal.ppat.1012283 (PMC11734982; doi:10.1371/journal.ppat.1012283)
Supplement: S5 Fig — (A-C) Mean and Standard Error data for Fig 7A–7C respectively. (D) Representative images analysed using the Incucyte software to obtain the: (i-vi) GFP object count and MFI data at the point of harvest for Fig 7A and Fig 7B ΔP1 wt, C11, ΔPK34, ΔPK234 and C11 ΔPK1234 GFP replicons and cell only control; and peak GFP object count data for Fig 7C (vii-xii) ΔP1 wt, C11, ΔPK34, ΔPK234 and C11 ΔPK1234 GFP replicons and transfected replicon only control. (PDF) [file ppat.1012283.s005.pdf]

S5 Fig.

wt			C11			ΔPK34			ΔPK234		
Mean	Standard deviation	Number	Mean	Standard deviation	Number	Mean	Standard deviation	Number	Mean	Standard deviation	Number
20724.2	345.9574	12	23063.6	412.21	12	21789.2	812.7529	12	23044	735.311	12

C11 ΔPK1234			Cell only		
Mean	Standard deviation	Number	Mean	Standard deviation	Number
15690.6	1186.97	12	0.6	0.4	12

(A) Mean and Standard Error data for Fig 7A.

wt			C11			ΔPK34			ΔPK234		
Mean	Standard deviation	Number	Mean	Standard deviation	Number	Mean	Standard deviation	Number	Mean	Standard deviation	Number
10.34976	0.357581	12	10.11416	0.287783	12	9.870948	0.375719	12	9.840355	0.23855	12

C11 ΔPK1234			Cell only		
Mean	Standard deviation	Number	Mean	Standard deviation	Number
6.846632	0.18476	12	4.844667	0.883832	12

(B) Mean and Standard Error data for Fig 7B.

wt			C11			ΔPK34			ΔPK234		
Mean	Standard deviation	Number	Mean	Standard deviation	Number	Mean	Standard deviation	Number	Mean	Standard deviation	Number
8500.467	101.7124	15	3416.267	119.1537	15	2547.333	73.72663	15	3304.8	119.5204	15

C11 ΔPK1234			Transfected replicon only		
Mean	Standard deviation	Number	Mean	Standard deviation	Number
294.3333	17.70571	15	7.8	3.184337	15

(C) Mean and Standard Error data for Fig 7C.

i. Fig 7A/B *wt* replicon R1 5 hrs

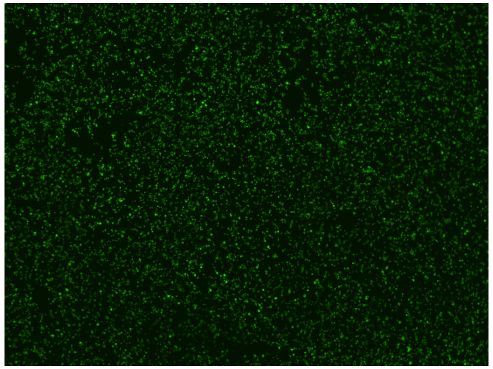

ii. Fig 7A/B C11 replicon R1 5 hrs

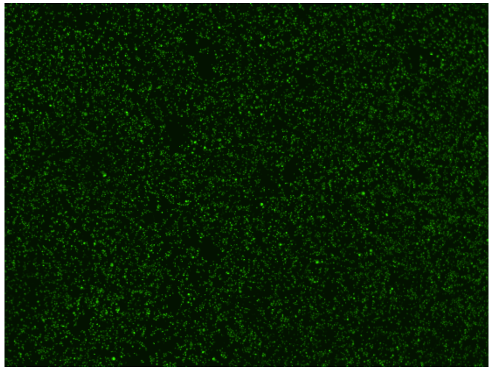

iii. Fig 7A/B ΔPK34 replicon R1 5 hrs

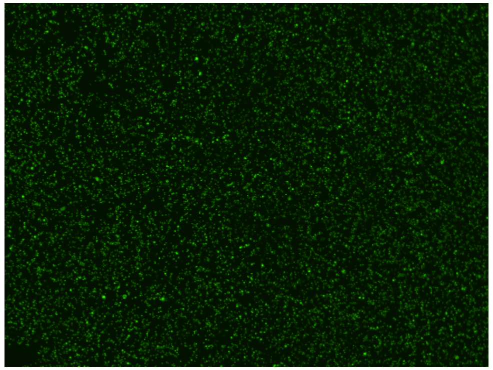

iv. Fig 7A/B ΔPK234 replicon R1 5 hrs

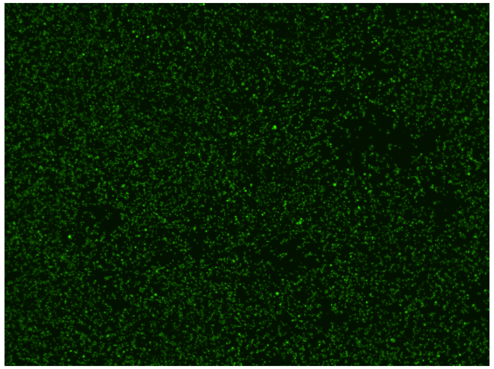

v. Fig 7A/B C11ΔPK1234 replicon R1 5 hrs

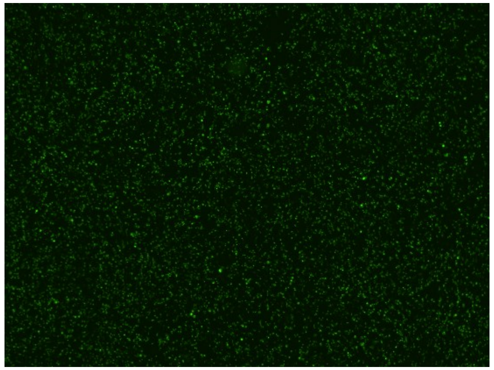

vi. Fig 7A/B cell only R1 5 hrs

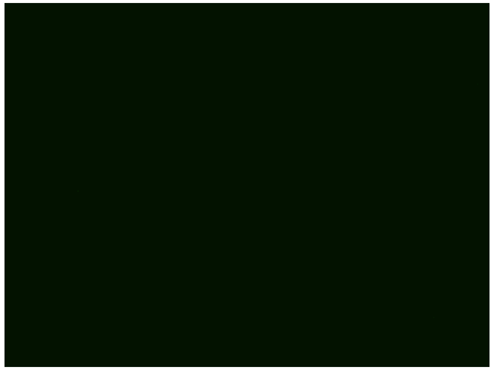

vii. Fig 7C *wt* replicon R2 8 hrs

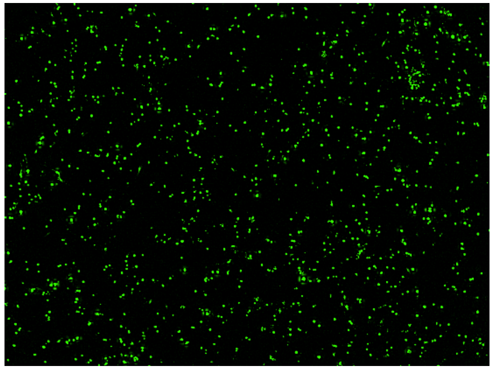

viii. Fig 7C C11 replicon R2 8 hrs

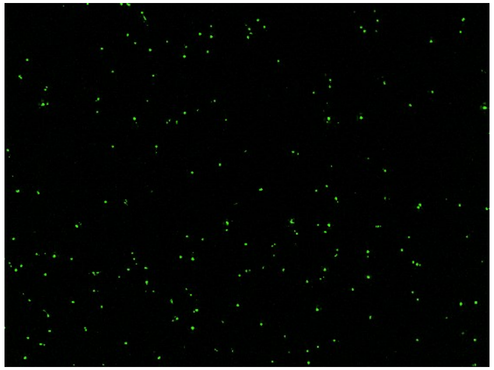

ix. Fig 7C ΔPK34 replicon R2 8 hrs

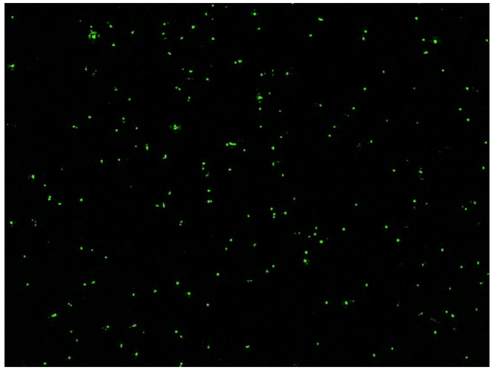

x. Fig 7C ΔPK234 replicon R2 8 hrs

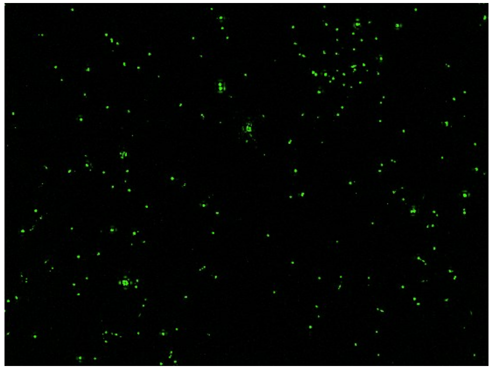

xi. Fig 7C C11ΔPK1234 replicon R2 8 hrs

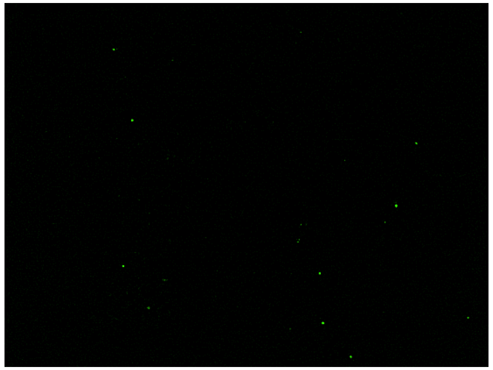

xii. Fig 7C transfected replicon only R2 8 hrs

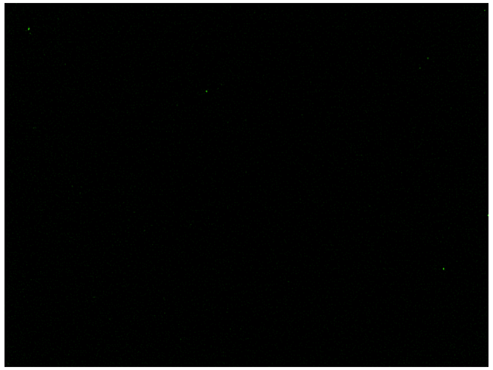

(D) Representative images analysed using the Incucyte software to obtain the: (i-vi) GFP object count and MFI data at the point of harvest for Fig 7A and Fig 7B ΔP1 *wt*, C11, ΔPK34, ΔPK234 and C11 ΔPK1234 GFP replicons and cell only control; and peak GFP object count data for Fig 7C (vii-xii) ΔP1 *wt*, C11, ΔPK34, ΔPK234 and C11 ΔPK1234 GFP replicons and transfected replicon only control.
