# Supplementary material for: The pseudoknot region and poly-(C) tract comprise an essential RNA packaging signal for assembly of foot-and-mouth disease virus
Source: PLoS Pathog. 2024 Dec 23;20(12):e1012283. doi: 10.1371/journal.ppat.1012283 (PMC11734982; doi:10.1371/journal.ppat.1012283)
Supplement: S6 Fig — (A-D) Mean and Standard Error data for Fig 8B–8E. (E) Representative images analysed using the Incucyte software to obtain the: (i-iv) GFP object count and MFI data at the point of harvest for Fig 8B and 8C wt, C39 ΔPK1 and C40 ΔPK2 ΔLbdcap GFP replicons and cell only control; peak GFP object count data for Fig 8D (v-viii) wt, C39 ΔPK1 and C40 ΔPK2 ΔLbdcap GFP replicons and transfected replicon only control; and (ix-xx) monolayer confluency for CPE measurement for wt, C39 ΔPK1 and C40 ΔPK2 ICs and cell only control at 0, 12 and 24 hrs. (PDF) [file ppat.1012283.s006.pdf]

S6 Fig.

wt			C39 ΔPK1			C40 ΔPK2			Cell only		
Mean	Standard deviation	Number	Mean	Standard deviation	Number	Mean	Standard deviation	Number	Mean	Standard deviation	Number
9998	1311.962	12	11631	1098.515	12	12390	1230.415	12	0.6	0.6	12

(A) Mean and Standard Error data for Fig 8B.

wt			C39 ΔPK1			C40 ΔPK2			Cell only		
Mean	Standard deviation	Number	Mean	Standard deviation	Number	Mean	Standard deviation	Number	Mean	Standard deviation	Number
5.300127	0.079471	12	5.330166	0.09233	12	5.855848	0.093446	12	4.226059	0	12

(B) Mean and Standard Error data for Fig 8C.

wt			C39 ΔPK1			C40 ΔPK2			Transfected replicon only		
Mean	Standard deviation	Number	Mean	Standard deviation	Number	Mean	Standard deviation	Number	Mean	Standard deviation	Number
8062.667	269.4118	12	3484.917	117.986	12	10242.33	372.8222	12	2.166667	2.166667	12

(C) Mean and Standard Error data for Fig 8D.

Time (hours)	*wt*			C39 ΔPK1			C40 ΔPK2			Transfected replicon only		
	Mean	Standard deviation	Number	Mean	Standard deviation	Number	Mean	Standard deviation	Number	Mean	Standard deviation	Number
0	82.99874	7.632228	12	85.13335	5.265405	12	82.4399	5.926969	12	82.57877	6.237126	12
1	80.20771	8.972347	12	83.79966	5.688993	12	79.51019	6.815244	12	82.75207	6.274942	12
2	77.37421	10.34624	12	83.22228	6.558325	12	76.48122	7.71649	12	85.59924	5.560315	12
3	74.10343	11.49497	12	81.60595	7.308624	12	72.79742	8.430058	12	88.15997	5.180708	12
4	71.89919	11.87037	12	79.32464	7.953166	12	70.24992	9.065388	12	90.56252	4.709153	12
5	70.43155	12.48139	12	77.02575	8.431832	12	68.22581	9.33341	12	92.22283	4.385059	12
6	69.31918	12.77925	12	75.46505	8.420245	12	66.95653	9.778826	12	93.71125	4.006099	12
7	68.47855	13.29392	12	74.04079	9.071277	12	65.92973	10.0814	12	94.64104	3.653795	12
8	67.63747	13.6962	12	73.20339	9.344368	12	64.92677	10.71402	12	95.65719	3.182549	12
9	66.70721	14.06123	12	72.44056	9.49233	12	63.55069	10.75593	12	96.41999	2.853242	12
10	65.66036	14.52489	12	71.92028	9.710411	12	62.20186	11.17988	12	97.05773	2.508587	12
11	64.56435	15.06407	12	70.6275	9.798356	12	61.13469	11.66187	12	97.6918	2.096464	12
12	63.80858	15.32577	12	70.46336	10.04694	12	60.07404	12.17533	12	98.13217	1.766882	12
13	62.92398	15.78827	12	69.99149	10.21109	12	59.00864	12.52297	12	98.60271	1.353087	12
14	61.8918	15.9059	12	69.7578	10.69097	12	57.71889	12.82027	12	98.96755	1.147901	12
15	60.82502	16.67423	12	69.07888	11.11414	12	56.86787	13.19038	12	99.2168	0.851103	12
16	59.71824	16.76435	12	68.39262	11.31407	12	55.87133	13.42815	12	99.43924	0.670856	12
17	58.68843	16.97823	12	68.17854	11.35601	12	54.51791	13.66481	12	99.56307	0.584663	12
18	57.38651	17.5565	12	67.77799	11.98986	12	53.60355	13.96184	12	99.65611	0.486796	12
19	56.06706	17.52262	12	67.19628	12.35386	12	52.27401	14.24326	12	99.7401	0.383166	12
20	54.6171	17.65573	12	67.08532	12.52884	12	51.37901	14.35968	12	99.76432	0.374011	12
21	53.29954	17.56185	12	66.46978	12.92326	12	50.30748	14.58714	12	99.82731	0.235436	12
22	51.94228	17.62849	12	66.39272	13.29293	12	49.26332	14.53102	12	99.8595	0.225549	12
23	50.25336	17.50418	12	65.76388	13.53131	12	48.33449	14.64205	12	99.87685	0.234314	12
24	48.75073	17.22027	12	65.05312	14.0508	12	46.89232	14.28707	12	99.90101	0.188394	12
25	47.33304	16.89638	12	64.45438	14.2674	12	46.10234	14.08492	12	99.91559	0.146898	12
26	46.04914	16.25816	12	63.96296	14.46505	12	44.83403	13.86885	12	99.92175	0.148003	12
27	44.47839	15.94357	12	63.13881	14.65611	12	43.80882	13.55129	12	99.93761	0.144907	12
28	43.0596	15.32171	12	62.57598	15.05317	12	42.75389	12.99281	12	99.94446	0.090379	12
29	41.66353	14.69142	12	61.75819	15.25905	12	41.74619	12.60605	12	99.94984	0.093446	12
30	40.6588	14.024	12	60.99007	15.47749	12	41.19654	12.64752	12	99.96417	0.054557	12
31	39.52122	13.32652	12	60.33698	15.74691	12	40.14536	12.0342	12	99.95941	0.059143	12

32	38.49709	12.61045	12	59.22122	15.86729	12	39.2039	11.60098	12	99.95882	0.043399	12
33	37.54905	11.88478	12	58.36575	16.34414	12	38.33622	11.0211	12	99.96711	0.045189	12
34	36.46868	11.03312	12	57.28256	16.48798	12	37.73137	10.81602	12	99.97482	0.032834	12
35	35.66114	10.30455	12	56.20692	16.3075	12	36.84818	10.32766	12	99.97584	0.03529	12
36	34.92356	9.692547	12	55.21463	16.34571	12	36.1722	9.898843	12	99.97831	0.036037	12
37	34.40857	9.09234	12	53.90239	16.26993	12	35.42397	9.622518	12	99.97843	0.040344	12
38	33.91664	8.535188	12	52.97227	16.06271	12	34.99813	9.2518	12	99.98087	0.035503	12
39	33.55413	8.232114	12	51.62079	15.78291	12	34.19127	8.79659	12	99.98171	0.025649	12
40	33.22031	7.80174	12	50.68797	15.57654	12	33.8031	8.397165	12	99.97546	0.034026	12
41	32.9256	7.503995	12	49.66934	15.13801	12	33.3098	8.150999	12	99.9808	0.025701	12
42	32.57944	7.19178	12	48.56921	14.83884	12	32.98666	7.802842	12	99.98151	0.024846	12
43	32.45506	6.899647	12	47.57686	14.48337	12	32.71562	7.454307	12	99.98046	0.029621	12
44	32.22156	6.730429	12	46.47338	14.06672	12	32.22461	7.165114	12	99.97994	0.045887	12

(D) Mean and Standard Error data for Fig 8E.

i. Fig 8B/C *wt* replicon R1 7 hrs

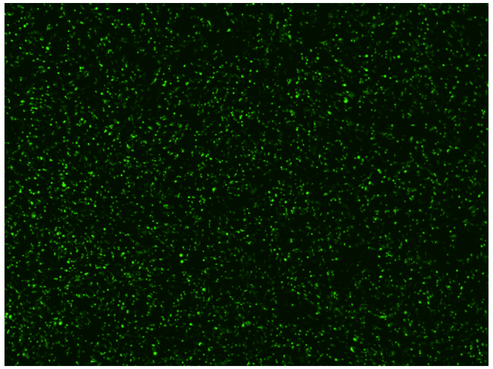

ii. Fig 8B/C C39 ΔPK1 replicon R1 7 hrs

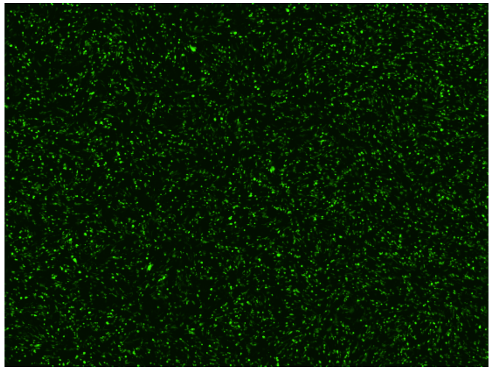

iii. Fig 8B/C C40 ΔPK2 replicon R1 7 hrs

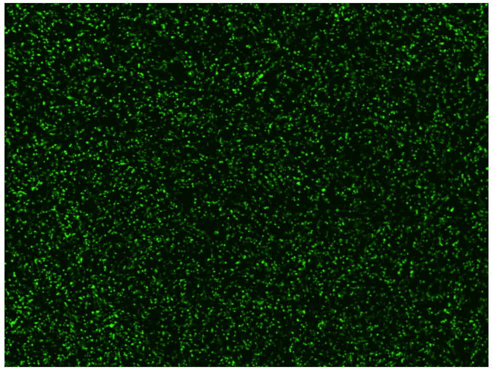

iv. Fig 8B/C cell only R1 7 hrs

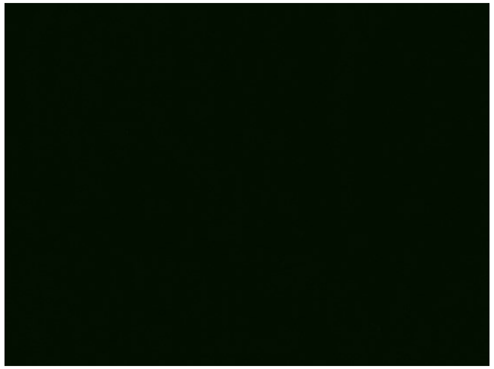

v. Fig 8D *wt* replicon R2 16 hrs

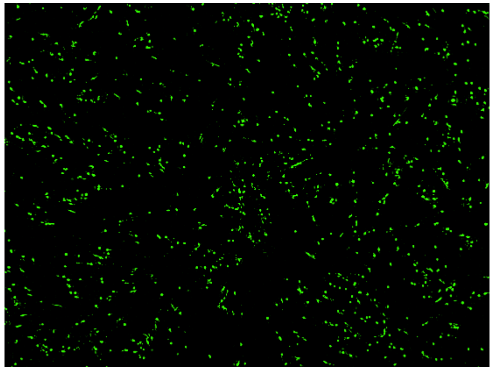

vi. Fig 8D C39 ΔPK1 replicon R2 16 hrs

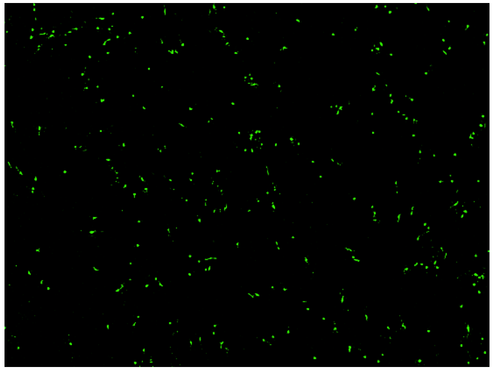

vii. Fig 8D C40 ΔPK2 replicon R2 16 hrs

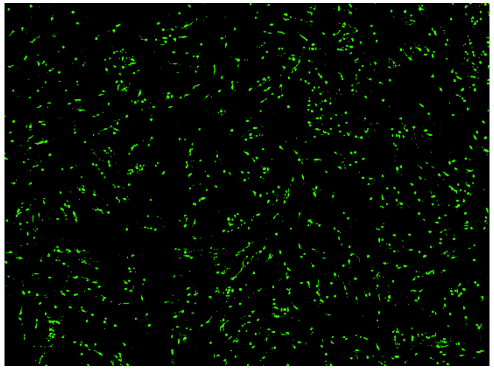

viii. Fig 8D transfected replicon only R2 16 hrs

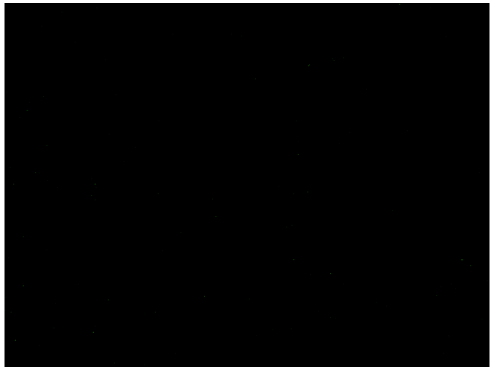

ix. Fig 8E *wt* IC 0 hrs

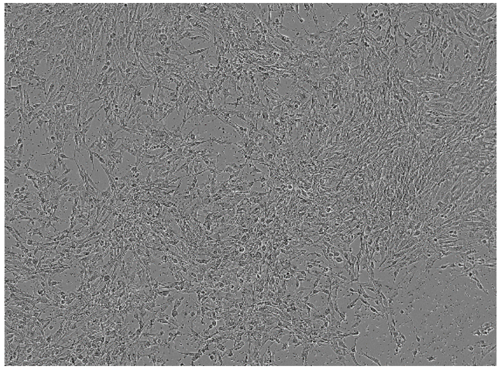

x. Fig 8E *wt* IC 12 hrs

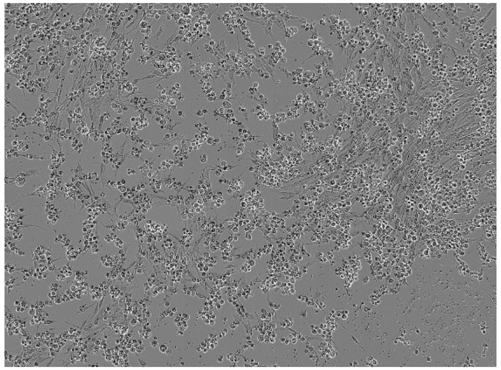

xi. Fig 8E *wt* IC 24 hrs

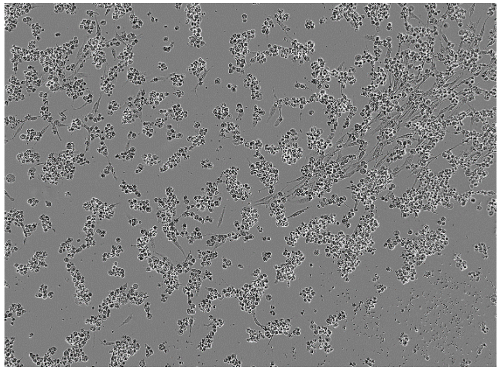

xii. Fig 8E C39 ΔPK1 IC 0 hrs

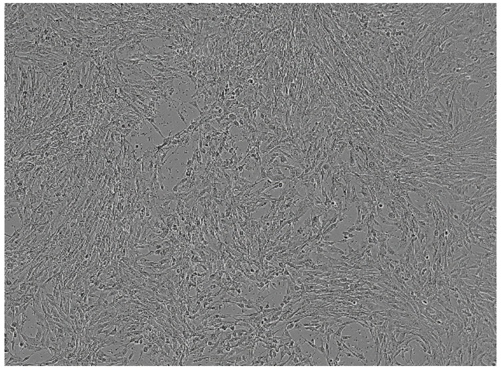

xiii. Fig 8E C39 ΔPK1 IC 12 hrs

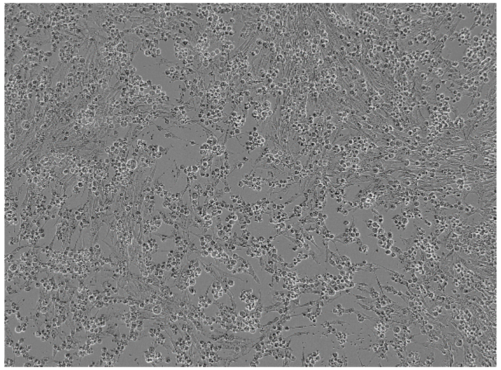

xiv. Fig 8E C39 ΔPK1 IC 24 hrs

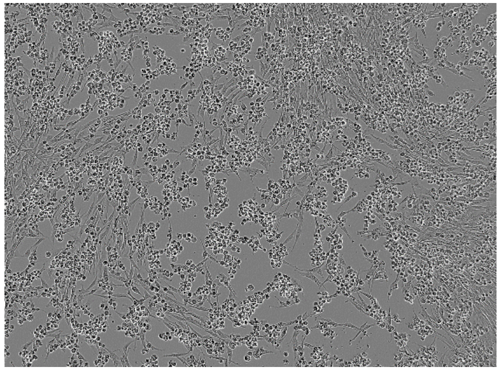

xv. Fig 8E C40 ΔPK2 IC 0 hrs

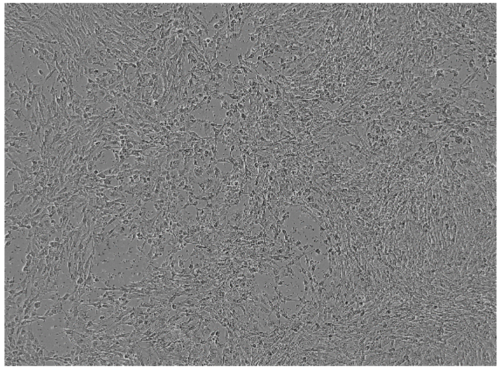

xvi. Fig 8E C40 ΔPK2IC 12 hrs

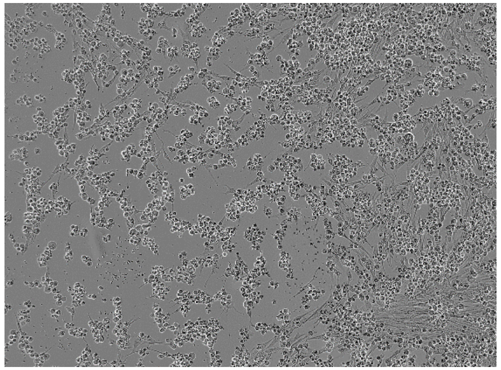

xvii. Fig 8E C40 ΔPK2IC 24 hrs

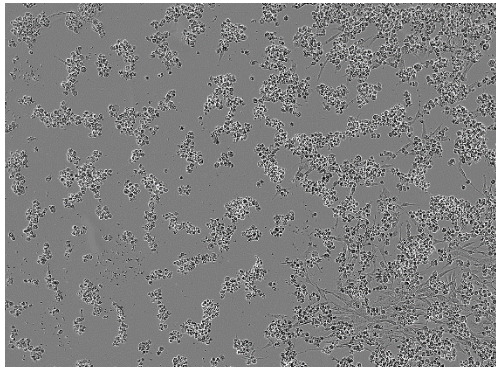

xviii. Fig 8E cell only IC 0 hrs

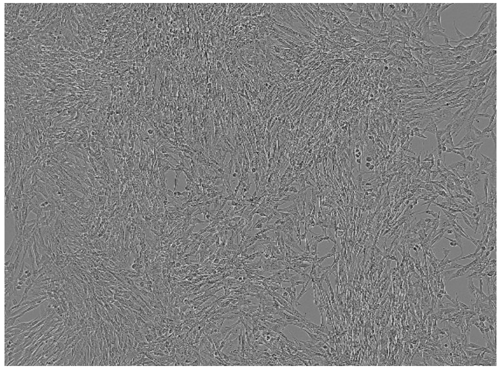

xix. Fig 8E cell only IC 12 hrs

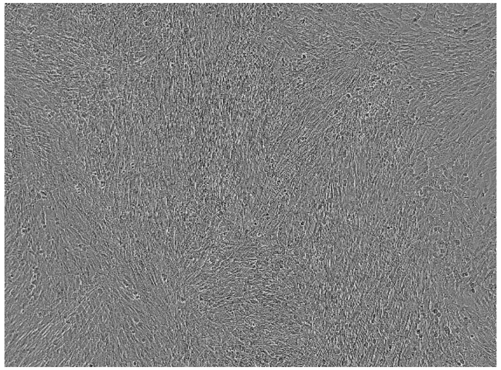

xx. Fig 8E cell only IC 24 hrs

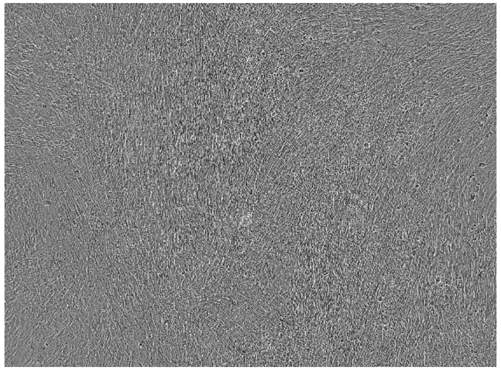

(E) Representative images analysed using the Incucyte software to obtain the: (i-iv) GFP object count and MFI data at the point of harvest for Fig 8B and Fig 8C *wt*, C39 ΔPK1 and C40 ΔPK2 ΔLbdcap GFP replicons and cell only control; peak GFP object count data for Fig 8D (v-viii) *wt*, C39 ΔPK1 and C40 ΔPK2 ΔLbdcap GFP replicons and transfected replicon only control; and (ix-xx) monolayer confluency for CPE measurement for *wt*, C39 ΔPK1 and C40 ΔPK2 ICs and cell only control at 0, 12 and 24 hrs.
