# Supplementary material for: The pseudoknot region and poly-(C) tract comprise an essential RNA packaging signal for assembly of foot-and-mouth disease virus
Source: PLoS Pathog. 2024 Dec 23;20(12):e1012283. doi: 10.1371/journal.ppat.1012283 (PMC11734982; doi:10.1371/journal.ppat.1012283)
Supplement: Attachment — Submitted filename: NeiletalPlosPathReviewer comments_responses_clean.docx [file ppat.1012283.s007.docx]

Dear reviewers,

Thank you for your feedback on our manuscript. Your comments have been useful, and in this document we have answered each of your queries individually and described the changes we have made as a result. We have submitted a manuscript with tracked changes on so these changes can be identified more easily, and we have listed the changes to the manuscript which are relevant to each comment in our responses below. Please note that the pages and line numbers referenced here correspond to the version of the manuscript *without* tracked changes.

Best regards,

Chris Neil

Reviewer #1

In the manuscript submitted by Chris Neil and Joseph Newman et al., the authors use two types of replicon: ΔP1 GFP replicon and ΔLbdcap GFP replicon to investigate FMDV RNA packaging through a novel trans-encapsidation assay, and reveal the essential role of packaging signals in FMDV RNA packaging, specifically those in the poly-(C) tract and pseudoknot (PK) region, where the length of the poly-(C) tract and PK1 influence the efficiency of RNA encapsidation. Based on this, they propose a simple model for FMDV RNA packaging, which involves a transition from genome replication to genome packaging and is controlled by packaging signals. These found could provide scientific guidance for the research and development of new antiviral strategies targeting FMDV and other picornaviruses.

1. The author transfected approximately 8kb of replicon RNAs such as C11, ΔPK34, ΔPK234, and C11 ΔPK1234 into BHK-21 cells. These different replicon mutants would alter the RNA structure and potentially alter the stability of the RNA in the cell. The author also needs to investigate whether the stability of these transfected RNAs in cells has changed.

- We have produced additional experimental data to look at both RNA stability and replication levels in cells using RT-qPCR, these data are presented in the new supplementary figure 2. In this experiment, we looked at the relative quantities of viral RNA present at 15 minutes and 6 hours post-transfection, in the presence or absence of an RNA replication inhibitor (guanidine hydrochloride, GuHCl). This was undertaken using the *wt*, ΔPK34, ΔPK234 and C11 ΔPK1234 replicons, as these incorporated the most drastic changes to the RNA, and the other mutations used in the manuscript were aimed at specific aspects of the modifications in these constructs. We found no significant differences in the Ct values of the mutant constructs compared to the *wt* construct after 6 hours in the presence or absence of the replication inhibitor (p < 0.005).

Changes in the manuscript:

- Line 441-443: Added a line describing the comparable stability of the RNA transcripts and referencing supplementary figure 2.
- Lines 261-266: Added the methods section for the RT-qPCR analysis
- Added supplementary figure 2 and the accompanying legend.

2. Whether replicons such as C11, C29, Δ PK234, and C11 Δ PK1234 have the same replication ability in cells, because the stronger the replication ability of the replicon RNA, the more RNA is replicated, and the higher the probability of being packaged. Therefore, in the second round of infection, the more GFP object count is present.

- The reviewer is correct in saying that replicons with greater RNA replication abilities would have more chance to be packaged. We have controlled for this by looking at GFP expression in the first round of the assay. This GFP expression is dependent on both RNA replication and translation, and an increase/decrease in either aspect would affect the GFP MFI. For every *trans*-­encapsidation assay we have performed, we have included this first round GFP expression data to check for differences in replicon transfection efficiency and replication ability.
- The only construct in which we observed a decrease in GFP expression in the first round was the C11 ΔPK1234 replicon, which is consistent with our previous publication (Ward, Lasecka-Dykes et al. 2022). To verify that this reduced GFP expression was due to a difference in RNA replication and not RNA stability, we performed RT-qPCR assays following incubation with or without GuHCl (an RNA replication inhibitor) to assess the intracellular stability of the RNAs, as explained in response to the first comment (supplementary figure 2). We found that the Ct values of all the constructs (aside from C11 ΔPK1234) were comparable to *wt* after 6 hours of replication, consistent with the equivalent GFP expression we observed. However, the Ct value for C11 ΔPK1234 was greater than *wt*, indicating that less RNA was present and therefore RNA replication was impaired, again consistent with the reduced GFP expression we observed.
- We have also tried to clarify this in the text by explaining how we used GFP expression in the first round as a proxy for RNA replication and translation.

Ward, J. C., et al. (2022). "The RNA pseudoknots in foot-and-mouth disease virus are dispensable for genome replication, but essential for the production of infectious virus." PLoS Pathog **18**(6): e1010589.

Changes in the manuscript:

- Lines 313-317, 358-359, 453-454: Expanded the text here to explain the use of GFP replication as a proxy for replicon replication.
- Lines 457-459: Reported the results of the RT-qPCR assays and added a reference to supplementary figure 2.
- Added supplementary figure 2 and the accompanying legend.

3. There is no difference between GFP and MFI in Figures 5a-b, which only indicates that they are similar in translation level and does not necessarily mean that there is no difference in replicon replication ability.

- GFP expression is dependent on both RNA replication and translation, and therefore we have used this as a proxy for general replicon replication ability. As with the second comment, we have tried to clarify this in the text. In addition, we have provided RT-qPCR data for the construct (C11 ΔPK1234), which had lower GFP expression in the first round (supplementary figure 2), to show that it had decreased RNA replication. We have also provided data on GFP expression in the presence of the replication inhibitor GuHCl (supplementary figure 1A). As RNA does not replicate in the presence of GuHCl the GFP signal is entirely dependent on translation of input RNA. When GuHCl is present, the GFP MFI is greater than that of the autofluorescence (in the cell only control), but less than that of fully competent replicons, showing that GFP expression reflects RNA replication levels and not just translation.

Changes in the manuscript:

- Lines 320-325: Added a sentence referencing S1 Fig and reporting the comparable translation-only GFP expression levels alongside the first *trans*-encapsidation assay described in the manuscript.
- Added S1 Fig and the accompanying legend.

4. In Figure 5E, the author assesses the trans-encapsidation efficiency of the C11, C29, and C35 (WT) genome donor replicon through using C11, C29, and C35 (WT) capsid donor infectious copy transcripts, respectively (lines 355-361). How can the author determine whether GFP comes from the capsid donor infectious copy transcripts or genome donor replicon? Is it possible that the difference in R2 Peak GFP object count is due to the difference in virus rescue between C11, C29, and C35 (WT) capped donor infection copy transcripts.

- The infectious copy plasmids we used as capsid-donors did not encode GFP, and therefore the only GFP signal present is derived from the replicons (genome donors). To make the infectious copy versions, we inserted the poly-(C) and PK mutations into the *wt* infectious copy plasmid which lacked the GFP coding region. We have clarified this in the text.

Changes in the manuscript:

- Lines 372-374: Added a sentence to clarify that the newly generated infectious copy plasmids do not encode GFP and hence it is only *trans*-encapsidated genomes that are detected.

5. The author used RNase T1 to digest transcripts and recovered RNA, but the recovered viral RNA contained a large amount of cellular RNA. Therefore, the digested "recovered multi C region 70" marked by the author in Figure 6B may mostly be cellular RNA, and it is recommended that the author add a cell group as a control.

- We agree and have now included the cell only control in the figure.

Changes in the manuscript:

- Line 855: Described the cell only control in the figure legend.
- Revised Fig 6.

6. Based on previous research, the author found that PK does not affect virus replication, but is it possible that C39 Δ PK1 and C40 Δ PK2 affect virus translation, leading to differences in GFP object and MFI？

- We have not looked in detail at how the PKs affect translation as this was not the focus of our study. We have used GFP expression as a proxy for RNA replication and translation. As the reviewer commented, it is possible that the increase in GFP MFI observed for C40 ΔPK2 was due to better translation efficiency, but this was not investigated further.

Changes in the manuscript:

- Line 514-515, 572-575: Added text referencing the caveat that the increase in R2 GFP count may be caused by the increase in general replication ability in the first round.

7. In Figure 8, C39 Δ PK1 and C40 Δ PK2 are described, while in the schematic diagram of Figure 1, C40 Δ PK1 and C39 Δ PK2 are labeled, leading to confusion in the experimental results.

- This was a nomenclature error on our part which has now been corrected. To clarify, the correct terms are C39 ΔPK1 and C40 ΔPK2.

Changes in the manuscript:

- Nomenclature of these constructs has been checked and corrected throughout, including Figure 1.

8. Suggest the author to use replication deficient viruses and replicons as controls to better elucidate the role of packaging signals in virus assembly.

- We evaluated the assay by performing the first round in the presence of the replication inhibitor GuHCl, and have now included these data in supplementary figure 1B. The presence of GuHCl in the first round inhibited *trans*-encapsidation, which is consistent with previous reports that only replicating RNA is able to be packaged.

Changes in the manuscript:

- Lines 329-331: Added a sentence describing the results of the *trans*-encapsidation assay when the first round was carried out in the presence of GuHCl and referring to supplementary figure 1.
- Lines 198-200: Added information regarding the use of GuHCl in the methods.
- Added supplementary figure 1 and the accompanying legend.

Reviewer #2

In this well-written manuscript, the authors describe a well-crafted study aimed at the long-standing question of how picornaviruses, positive-sense RNA viruses, package their genomic RNAs into virion capsids. Unlike other viruses, e.g., retroviruses, there has been no description of a single RNA sequence or structural element that directs newly-synthesized viral RNAs into newly-formed capsid structures. In the present study, that authors have chosen to address this question using FMDV as their model picornavirus, a virus that is known to infect cloven hooved animals and cause major outbreaks. The authors showed that specific RNA sequence/structure elements [including pseudoknots and the poly(C) tract in the 5’ UTR] dispersed throughout the genome play important roles in RNA packaging for FMDV. Using a clever replicon rescue, trans-encapsidation/RNA packaging assay, they identified several packaging sequences and showed that the length of the poly(C) tract impacts encapsidation, with longer tracts producing higher levels of packaging efficiency. In these studies, the authors were particularly careful to eliminate other potential variables/contributors to solidify their conclusions, using a two round assay to their advantage. This led them to propose a very plausible model to explain their data, although the model may not fit for enteroviruses like poliovirus. With one exception (see below) the Introduction and Discussion sections were thoughtfully written.

1. The authors have omitted a discussion of the data provided by Song and co-workers in their 2017 PNAS paper (PMID: 28973853) describing their unsuccessful search for RNA sequences/structures required for poliovirus genome encapsidation using massive genome sequence recoding. Although a much different picornavirus than FMDV, poliovirus packaging stands in contrast to what the authors have proposed in Figure 9, and the last paragraph of the Discussion does not accurately reflect the inclusion of the data from the Song et al. study. The fact that this different genus of picornaviruses has not been shown to contain one or more RNA packaging signals (despite an exhaustive recoding analysis) that guide RNA encapsidation suggests the possibility of additional signals (RNP complexes?) playing a role in encapsidation. The Discussion of the revised manuscript should contain additional consideration of this topic.

- We have updated the discussion to reflect this. Although we acknowledged the model which does not involve packaging signals, we have now specifically mentioned the recoding study. While we acknowledge that Song et al. suggested a lack of packaging signals in PV, it is important to note that Chandler-Bostock et al. (Chandler-Bostock, Mata et al. 2020) provided evidence for packaging signals dispersed throughout the genome for enteroviruses, including PV. We reference both points of view and try to accommodate both in our model; protein-protein interactions are undoubtedly important, but RNA-protein interactions are also present. However, we appreciate that the relative roles of these postulated packaging mechanisms may differ among the picornaviruses.

Chandler-Bostock, R., et al. (2020). "Assembly of infectious enteroviruses depends on multiple, conserved genomic RNA-coat protein contacts." PLoS Pathog **16**(12): e1009146.

Changes in the manuscript:

- Lines 649-661: Added discussion referencing Song et al and Chandler-Bostock et al. specifically.

2. Line 140: The Introduction section could use a better concluding sentence stating what the study actually showed, not just to “characterize.”

- We agree and have expanded this final sentence

Changes in the manuscript:

- Lines 139-141: Updated the concluding sentence for the introduction.

*We then used this assay to demonstrate the importance of both the poly-(C) tract and the neighbouring PSs in the PK region with regards to RNA packaging and propose a potential model for FMDV packaging that relies on the PSs dispersed across the genome.*

3. Lines 246 and 250: Manufacturer guidelines, although a popular cop out, is not sufficient here – please describe what was done.

- We have added more details regarding the protocols used for this section

Changes in the manuscript:

- Lines 249-251, 253-259: Expanded on the methods used.

4. Figure 4: Why no apparent heterogeneity in the poly(C) tract here?

- The figure referenced assessed the poly-(C) tract lengths in the plasmid, so we would not expect heterogeneity here as the plasmids should all contain uniform poly-(C) tract lengths. We have tried to clarify this further in the text that these are DNA fragments from plasmids.

5. For the experiment described at the bottom of page 17, why not use RNase treatment of the recovered virus yields following the first round transfection? This would insure no carryforward of RNAs.

- We considered this, but were ultimately averse to treating our samples with RNase. Even though the RNA would be purified, we were concerned that residual traces of the RNase would affect the RNA and so we used a single passage to separate the packaged RNA away from the unpackaged and input RNA in the first round.

6. In Figure 6A, the resolution in the gel is not high enough to make a strong conclusion. Also, the experimental details of automated electrophoresis should be expanded here or in the figure legend so the reader can actually know what was done.

- We appreciate that the resolution here is not sufficient to put exact numbers on the length of the poly-(C) tract, but we believe that these data demonstrate the difference between the poly-(C) tract lengths of the viruses before and after recovery, which is the main point we want to convey. We have amended the text to acknowledge this, putting less emphasis on the exact size of the peak and describing the peaks as being broad and therefore not representing a single uniform poly-(C) tract length. We have also added more detail to the methods to better explain the electrophoresis protocol.

Changes in the manuscript:

- Lines 420-421, 423-428: Added descriptions regarding the broadness of the peaks and acknowledging the caveat that there is no defined length poly-(C) tract and instead there is a range of different sizes around the quoted length.

7. Line 453: The authors need to independently assess the poly(C) tract length and not depend on what the manufacturer states – they are not co-authors of this manuscript.

- We were not able to sequence these constructs due to the difficulties of sequencing through the poly-(C) tract, but we did analyse these constructs using the Tapestation and electrophoresis as we did for the other poly-(C) tract constructs to verify that the plasmid fragments containing the poly-(C) tract and PK region were of the expected size. These data have now been added to Figure 8.

Changes in the manuscript:

- Lines 494-499: Added text referencing Fig 8F and the verification of the constructs, explaining the rationale behind the approach as opposed to sequencing.
- Added panel F to Figure 8 to show the Tapestation analysis of the plasmid fragments containing the poly-(C) tract and PK region, as was done with the poly-(C) tract mutants in Fig 4.

8. For the experiments described in the last paragraph of page 21, were the complete genomic sequences determined for the RNAs of the recovered viruses? This is a key question, since there may be lesions outside of the targeted regions of the FMDV genome that contribute to the phenotypes observed by the authors.

- We have not sequenced the recovered viruses in this study. In our previous publication (Ward, Lasecka-Dykes et al. 2022), where we generated the replicons and viruses with sequential PK deletions (all of which were larger than the deletions described here), we did passage and sequence those viruses and found no changes after four passages. Consequently, given the speed at which the phenotype was observed after transfection and the correlation of the phenotype of the viruses with the *trans*-encapsidation assay, we did not sequence these viruses as we did not expect to see any variants. We agree it would be interesting to investigate whether changes to the PK region occur over several passages, however, but we have not undertaken these experiments.

Ward, J. C., et al. (2022). "The RNA pseudoknots in foot-and-mouth disease virus are dispensable for genome replication, but essential for the production of infectious virus." PLoS Pathog **18**(6): e1010589.

9. Line 507: What is a “robust” method? Although it is common to (mis)use this word in scientific vernacular, it really is inappropriate here.

- We have replaced “robust” with “reproducible”

Changes in the manuscript:

- Line 554: Updated the terminology used

10. In the Discussion on pages 26 and 27 (with reference to the model in Figure 9), additional considerations should be discussed. If there are two pathways (packaging and replication) for newly synthesized RNAs, how are they localized within the cytoplasm? For the RNAs in the replication pathway to be replicated, they must first be translated. Have the authors used imaging methodologies to track the newly-synthesized RNAs to distinct locations in the cytoplasm of infected cells (e.g., membrane-bound RNA replication complexes, packaging complexes, or translating ribosomes)? Even if these studies have not yet been carried out, the subcellular fate of newly synthesized FMDV RNAs should be discussed in the manuscript.

- In our proposed model, we did not intend to go into detail about the sub-cellular localisation of FMDV RNA as our focus was on packaging and to provide a hypothesis about how packaging could be regulated through packaging signals. As such, we have kept the concept simple and made the assumption that packaging, replication and translation all occur at sites that are at least close enough to each other to facilitate easy transport of RNA/proteins between each site.
- It has also been shown previously that only replicating RNA can be packaged for picornaviruses, which implies that there may be a link between the capsid proteins and the replication complex which can differentiate between replicating and non-replicating RNA, which is the basis of the assumption in the model that capsid proteins are able to interact with replicating RNA during assembly.
- It is of course also possible that the sites of replication and assembly are not completely co-localised, and instead there is transfer of RNA across to the capsid proteins and the site of assembly. But for the sake of simplicity when proposing this hypothesis, and because we do not have information about exactly where the RNA and capsid proteins are located, we have adopted the most straightforward concept.

Chandler-Bostock, R., et al. (2020). "Assembly of infectious enteroviruses depends on multiple, conserved genomic RNA-coat protein contacts." PLoS Pathog **16**(12): e1009146.

Picornaviruses are important viral pathogens, but despite extensive study, the assembly process of their infectious virions is still incompletely understood, preventing the development of anti-viral strategies targeting this essential part of the life cycle. We report the identification, via RNA SELEX and bioinformatics, of multiple RNA sites across the genome of a typical enterovirus, enterovirus-E (EV-E), that each have affinity for the cognate viral capsid protein (CP) capsomer. Many of these sites are evolutionarily conserved across known EV-E variants, suggesting they play essential functional roles. Cryo-electron microscopy was used to reconstruct the EV-E particle at ~2.2 Å resolution, revealing extensive density for the genomic RNA. Relaxing the imposed symmetry within the reconstructed particles reveals multiple RNA-CP contacts, a first for any picornavirus. Conservative mutagenesis of the individual RNA-contacting amino acid side chains in EV-E, many of which are conserved across the enterovirus family including poliovirus, is lethal but does not interfere with replication or translation. Anti-EV-E and anti-poliovirus aptamers share sequence similarities with sites distributed across the poliovirus genome. These data are consistent with the hypothesis that these RNA-CP contacts are RNA Packaging Signals (PSs) that play vital roles in assembly and suggest that the RNA PSs are evolutionarily conserved between pathogens within the family, augmenting the current protein-only assembly paradigm for this family of viruses.

Ward, J. C., et al. (2022). "The RNA pseudoknots in foot-and-mouth disease virus are dispensable for genome replication, but essential for the production of infectious virus." PLoS Pathog **18**(6): e1010589.

Non-coding regions of viral RNA (vRNA) genomes are critically important in the regulation of gene expression. In particular, pseudoknot (PK) structures, which are present in a wide range of RNA molecules, have a variety of roles. The 5' untranslated region (5' UTR) of foot-and-mouth disease virus (FMDV) vRNA is considerably longer than in other viruses from the picornavirus family and consists of a number of distinctive structural motifs that includes multiple (2, 3 or 4 depending on the virus strain) putative PKs linked in tandem. The role(s) of the PKs in the FMDV infection are not fully understood. Here, using bioinformatics, sub-genomic replicons and recombinant viruses we have investigated the structural conservation and importance of the PKs in the FMDV lifecycle. Our results show that despite the conservation of two or more PKs across all FMDVs, a replicon lacking PKs was replication competent, albeit at reduced levels. Furthermore, in competition experiments, GFP FMDV replicons with less than two (0 or 1) PK structures were outcompeted by a mCherry FMDV wt replicon that had 4 PKs, whereas GFP replicons with 2 or 4 PKs were not. This apparent replicative advantage offered by the additional PKs correlates with the maintenance of at least two PKs in the genomes of FMDV field isolates. Despite a replicon lacking any PKs retaining the ability to replicate, viruses completely lacking PK were not viable and at least one PK was essential for recovery of infections virus, suggesting a role for the PKs in virion assembly. Thus, our study points to roles for the PKs in both vRNA replication and virion assembly, thereby improving understanding the molecular biology of FMDV replication and the wider roles of PK in RNA functions.
